# Population Genetic Study of *vitellogenin* in Honey Bees ( *Apis mellifera* ) With European Ancestry Identifies Two Ancestral Genetic Backgrounds

**DOI:** 10.1002/ece3.73845

**Published:** 2026-06-15

**Authors:** Vilde Leipart, Reed A. Cartwright, Adam Eyre‐Walker, Simen R. Sandve, Gro V. Amdam

**Affiliations:** ^1^ Faculty of Environmental Sciences and Natural Resource Management Norwegian University of Life Sciences Ås Norway; ^2^ Research Department of Structural and Molecular Biology University College London London UK; ^3^ School of Life Sciences Arizona State University Tempe Arizona USA; ^4^ The Biodesign Institute Arizona State University Tempe Arizona USA; ^5^ School of Life Sciences University of Sussex Brighton UK; ^6^ Faculty of Biosciences, Department of Animal and Aquacultural Sciences Norwegian University of Life Sciences Ås Norway

**Keywords:** *Apis mellifera*, genetic variation, haplotype structure, sequence polymorphisms, Vitellogenin

## Abstract

In honey bees ( *Apis mellifera* ), the ancient and multifunctional protein Vitellogenin (Vg) is tightly linked to colony health. Vg contributes to several key traits, including nutrient transport, immunity support, and regulation of social behavior. However, the role of selective forces in shaping natural variation in this gene is poorly understood. To address this, we use a population genetic approach based on long‐read sequences to characterize full‐length *vg* haplotype variation. Using sequences from 543 honey bees sampled in Europe and the USA, including different subspecies and multiple geographical locations and we test whether patterns of variation and divergence are compatible with selection. Our findings show that *vg* segregates into two main haplotype versions (haplogroups), defined by 65 common polymorphisms, which together account for 69% of the sampled haplotypes. One haplogroup is abundant in *A. m. mellifera* conservatory samples. At the protein level, the haplogroups differ at only seven positions, all of which are in the protein's lipid‐binding cavity. This concentration at a single functional region motivates the hypothesis that haplogroup differences may affect lipid binding or transport. The remaining 58 polymorphisms are synonymous or in noncoding regions, which could influence gene expression and splicing. We further find that comparisons of variation across our samples are compatible with a history of positive selection at *vg*, in line with prior work. Taken together, our results document substantial *vg* haplotype structure across sampled honey bees and identify protein‐coding differences concentrated in the lipid‐binding cavity. This provides a foundation for future work linking *vg* genetic variation to Vg function and colony‐related phenotypes.

## Graphical abstract

Our work shows that honey bee vitellogenin (Vg) segregates into two main haplotype versions (haplogroups) defined by 65 common polymorphisms. One haplogroup is abundant in A. m. mellifera conservatory samples, and at the protein level, we identify protein‐coding differences concentrated in the lipid‐binding cavity. This provides a foundation for future work linking vg genetic variation to Vg function and colony‐related phenotypes.

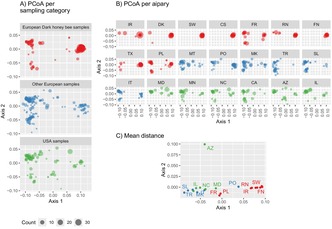

## 1 Introduction

Identifying candidate genes potentially shaped by selection and characterizing their genetic variation can help us understand how species adapt to their environments. The ecologically and economically important pollinator, the honey bee ( *Apis mellifera* ), is now distributed globally through historical range expansion and, more recently, managed introductions (Whitfield et al. 2006; Han et al. 2012; Wallberg et al. 2014; Visick and Ratnieks 2023). This biogeographic history reflects long‐term genetic differentiation among geographically distinct lineages (C in Europe; M in Europe and Asia; A, L and U in Africa; and O and Y in Asia (Wallberg et al. 2014; Chen et al. 2016; Dogantzis and Zayed 2019; Dogantzis et al. 2021)), and around 33 recognized subspecies (Ruttner 1988; Eimanifar et al. 2018; Ilyasov et al. 2020), with whole‐genome analyses providing temporal and demographic context for lineage divergence and secondary contact (Wallberg et al. 2014; Dogantzis et al. 2021; Leroy et al. 2024). These classifications were historically based on biogeography and morphometric characters (Ruttner 1988; Engel 1999) and are now increasingly evaluated and refined using genome‐wide single‐nucleotide polymorphism (SNP) markers (Wallberg et al. 2014; Eimanifar et al. 2018; Wragg et al. 2022), which are effective in measuring the level of introgression between lineages and subspecies (Pinto et al. 2014; Munoz et al. 2017; Henriques, Browne, et al. 2018). Several studies suggest that some of the native honey bee subspecies show evidence of adaptation to local environments, climates, and pathogens (Meixner et al. 2015; Henriques, Wallberg, et al. 2018; Schaumann et al. 2024; Parejo et al. 2025). These native subspecies represent an essential source of genetic variation that can be associated with adaptive traits (Pinto et al. 2014; Henriques, Wallberg, et al. 2018). Genome‐wide SNP scans have identified loci and genomic regions with signatures of positive selection in honey bees (Zayed and Whitfield 2008; Chávez‐Galarza et al. 2013; Harpur et al. 2014; Wallberg et al. 2014), and studies focusing on single genes have shown patterns consistent with positive selection in particular populations (Kent et al. 2011; Henriques, Wallberg, et al. 2018; Henriques et al. 2021). Characterizing variation at such candidate loci and genes can provide insight into how biological functions may be shaped by natural selection. For honey bees, such knowledge is timely and important because traits that contribute to adaptation may play roles in recent patterns of colony loss (Harpur et al. 2014; Brosi et al. 2017; Minaud et al. 2024). Current losses of managed honey bees are of international concern, driven by poor nutrition (DeGrandi‐Hoffman and Chen 2015; Dolezal and Toth 2018; Dolezal et al. 2019), changing weather conditions (Switanek et al. 2017; Cornelissen et al. 2019; Insolia et al. 2022; Zapata‐Hernández et al. 2024), as well as pests, pathogens, and pesticides (Cornelissen et al. 2019; Dolezal et al. 2019; Insolia et al. 2022).

Vitellogenin (Vg) is an ancient and multifunctional protein linked to several important traits central to honey bee fitness, and prior work has reported patterns consistent with positive selection at the *vg* locus (Kent et al. 2011). Vg is an essential protein in oviparous animals and the predominant lipoprotein found in the yolk of most egg‐laying species. It is a lipid transport protein and is necessary for reproduction by delivering nutrients, such as zinc and lipid molecules, to developing embryos (Tufail and Takeda 2008). In the honey bee, Vg has been identified as a key player in health and behavior. Vg can recognize pathogen surfaces and subsequently transport immune elicitors to larvae, thereby priming the offspring's immune system (Havukainen et al. 2013; Salmela et al. 2015). It also supports the maintenance of immune cells (Amdam et al. 2004) and protects against oxidative stress (Seehuus et al. 2006), increases longevity during overwintering (Amdam et al. 2005; Döke et al. 2015), and regulates complex social behavior (Amdam et al. 2003; Havukainen et al. 2011; Schilcher and Scheiner 2023). More recently, a Vg subunit has been shown to translocate into the nuclei of honey bee cells and interact with DNA (Salmela et al. 2022), thereby altering gene expression (Harwood et al. 2025). Similar multifunctionality is found for Vg in ants (Morandin et al. 2014), fish (Sun et al. 2013; Zhang et al. 2015), scallops (Wu et al. 2015), and nematodes (Perez and Lehner 2019).

Proteins like Vg that are involved in binding or cell‐surface interactions can show elevated rates of evolution, potentially reflecting changes in their binding partners (Diaz et al. 2018; Hilbert et al. 2023; Nandakumar et al. 2025). Changing binding partners might result from shifts in the local environment, such as pathogen communities, diets, or climate. Such ecological differences may be associated with differences in selective pressures that drive molecular evolution (Doublet et al. 2017; Henriques, Wallberg, et al. 2018; Sarioğlu‐Bozkurt et al. 2022). At the same time, essential and conserved functions in reproduction, as is the case for Vg, can impose strong evolutionary conservation (Bergmiller et al. 2012; Luo et al. 2015; Kay et al. 2025). Together, these perspectives motivate the hypothesis that different regions of Vg may experience different evolutionary pressures. For example, regions involved in immune support or lipid binding may evolve more rapidly, whereas regions essential for reproduction may remain more conserved. This may explain previous observations of both conserved and rapidly evolving domains in Vg (Leipart et al. 2022).

Previous work on SNP variation in the *vg* gene revealed patterns consistent with adaptive evolution among honey bee lineages (Kent et al. 2011) and highlighted amino acid chaining SNPs (non‐synonymous, nsSNP) associated with particular honey bee subspecies (Ilyasov et al. 2015). However, these studies relied on short sequences of individual *vg* exons from a few selected populations. They therefore did not capture intronic SNPs, full‐length gene variation, haplotypes (i.e., the unique combinations of all SNPs), or SNP variations across geographically diverse populations and subspecies.

We built on these exon‐based snapshots by assembling full‐length *vg* haplotypes using long‐read sequencing technology with individually barcoded primers. This sequencing approach provided complete coverage of the *vg* gene, collecting SNP variation from both coding and non‐coding gene regions. Barcoded long‐read sequences allowed us to reconstruct full‐length *vg* haplotype pairs for individual bees and identify coding and non‐coding variants that co‐occur on the same haplotype. The dataset includes *vg* sequences from 543 honey bees collected in Europe and the USA. In our initial study of this dataset (Leipart et al. 2022), we identified 81 nsSNPs across the protein, which were non‐uniformly distributed. The Vg protein regions encoding lipid‐binding cavity, responsible for transport of lipid molecules, were highly enriched with nsSNPs, while Vg regions encoding DNA and receptor‐binding site were conserved. Together, these patterns motivate the hypothesis that different functional regions of Vg may experience different evolutionary pressures. Building on this starting point, we now use the full‐length haplotype data to describe *vg* haplotype structure across sampled honey bees and to evaluate whether patterns of variation and divergence are compatible with selection.

Using the full‐length haplotype sequences, we identify two *vg* haplogroups, defined by two dominant haplotypes, with one more abundant in the European Dark honey bee samples. Differences between haplogroups include seven amino acid changes concentrated in the lipid‐binding cavity of Vg. This pattern is consistent with the possibility that Vg's lipid‐binding capabilities differ among haplotypes or, alternatively, that this region is more tolerant of amino acid changes. We also identify nucleotide changes in both exons and introns not altering the protein sequence or structure but may impact gene regulation or expression (Hane et al. 2016; Bampi et al. 2020). Comparison of variation within our samples to divergence from an outgroup further suggests patterns compatible with a balance between purifying constraints and adaptive evolution in this protein.

## 2 Results and Discussion

### 2.1 Strong Genetic Structures

Our dataset contained 1086 *vg* haplotype sequences from 543 honey bees, spanning 7 exons, 6 introns, and a total of 6094 bps, corresponding to the complete *vg* gene. Variant calling identified 397 biallelic sites in the dataset (Figure S1A) and 351 unique haplotypes (Figure S1B). Principal‐coordinate analysis (PCoA) on the distances between the haplotypes revealed a clear genetic structure (Figure S1C). The spread along the first axis suggests that there may be two main groups of related haplotypes or “haplogroups” in our data (Figure S1D).

### 2.2 Two Major Haplotype Groups at the *vg* Locus

**FIGURE 1:**
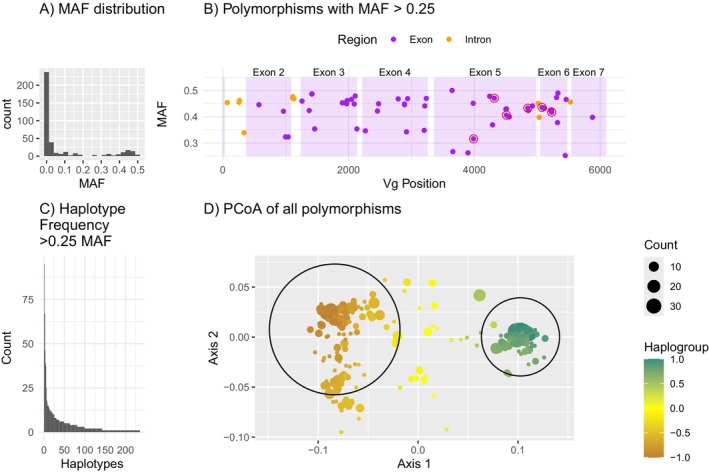
(A) Distribution of polymorphisms by MAF. (B) Distribution of identified polymorphisms > 0.25 MAF across the vg gene (x‐axis), colored by intron (yellow) or exon (purple) region. nsSNPs are marked with red circles. (C) The identified haplotypes (x‐axis) using only positions > 0.25 MAF, sorted by highest to lowest frequency (y‐axis). (D) Two‐dimensional PCoA of all haplotypes identified in Figure S1. Point size reflects haplotype abundance (see count legend). Color represents pairwise distance to the two most common haplotypes, defined by polymorphisms with MAF > 0.25, (see haplotype legend). Green (1.0) represents the most frequent haplotype (*n* = 95), and brown (−1.0) represents the second most frequent (*n* = 67). Haplogroups are highlighted with black circles and defined as haplotypes with pairwise distances < 0.2 to one of the two most common haplotypes.

We investigated ancestral genetic structure within our *vg* gene haplotype data. To minimize the influence of potentially recent (and rare) variation, which can obscure ancestral genetic patterns, we focused on the most common variants with a minor allele frequency (MAF) greater than 0.25, which, although chosen arbitrarily, does represent an observed break in our dataset (Figure 1A). This approach identified 65 variants distributed throughout most regions of the *vg* gene, except for exon 1, intron 3, and intron 4 (see Figure 1B and Table S1). These variants defined 235 distinct haplotypes (Figure 1C). Notably, the two most common haplotypes together account for 69% of the dataset (40% and 29%), indicating that they capture most of the variation. These two dominating haplotypes were fixed for different variants across all 65 polymorphic sites. PCoA of haplotype diversity revealed two distinct groups separated along Axis 1 (Figure 1D). Importantly, this structure was not an artifact of excluding rare alleles (Figure S2). The two haplotype groups (Axis 1, Figure 1D) clustered around the two most common haplotypes. Finally, we assigned 85% of the haplotypes to one of two haplogroups, haplogroup 1 (green) and haplogroup 2 (brown), using a Hamming distance cutoff < 0.2 relative to either of the two major haplotypes (see black circles in Figure 1D).

Honey bees are known to exhibit one of the highest recombination rates among animals (Beye et al. 2006; Solignac et al. 2007; Kent et al. 2012). This is important to consider for population genetic analysis since recombination can influence estimates of selection and genetic differentiation (Schierup and Hein 2000; Scheffler et al. 2006; Dumont 2020; Samuk et al. 2020). We evaluated whether any *vg* haplotypes in our dataset showed signs of recombination using the two most common haplotypes (*n* = 97 in haplogroup 1 and *n* = 67 in haplogroup 2) as standards. We classified haplotypes that contained fragments from both as putative recombinants (see methods for details) and identified 171 recombinant haplotypes. These are positioned between the two haplogroups along the first PCoA axis (Figure S3). Note that, due to the high recombination rate in the dataset, we did not find phylogenetic analyses particularly insightful for this study.

Next, we used the *F*_ST_ to estimate the overall genetic differentiation between all haplotypes, including and excluding the recombinant haplotypes. Between all haplotypes, we find that the global *F*_ST_ is 0.037, which is low (an *F*_ST_ < 0.05 indicates low genetic differentiation, 0.05–0.15 is intermediate, while > 0.25 is high (Hartl et al. 1997)). Excluding the recombinant haplotypes increases the global *F*_ST_ to 0.051 (Figure S4). This confirms our results from the PCoA analyses: the recombinant sequences are a mixture of the two haplogroups and, therefore, excluding them makes the genetic structure even more distinct.

### 2.3 Distinct Abundance of Haplogroups

Our dataset consists of haplotypes from three sampling categories of honey bees: European Dark honey bee collected from conservatories (*A. m. mellifera*), European honey bees collected from core breeding locations (*A. m. iberiensis, A. m. carnica, A. m.ligustica, A. m. macedonica, A. m.ruttneri*, and *A. m. anatolica*), and honey bees sampled from geographically distributed apiaries in the USA. These samples were assembled to map full‐length *vg* variation across hundreds of honey bees and to exemplify how the *vg* genetic variation can be distributed across different genetic backgrounds and locations. The first category of European Dark honey bees is expected to experience restricted gene flow, which allows for some assessment of within‐subspecies variation. This is due to these bees being classified as locally endangered (NordGen 2019), so native Dark bees have been sheltered in their environment by creating conservatory apiaries throughout Northern Europe, now existing for 20–30 years (De la Rúa et al. 2009; Pinto et al. 2014; Bertrand et al. 2015; Parejo et al. 2016; Munoz et al. 2017; Hassett et al. 2018; Henriques, Browne, et al. 2018). The second category includes a subset of individuals from other European subspecies to facilitate limited comparisons of *vg* variation across subspecies. The third category represents samples with mixed ancestry (Carpenter and Harpur 2021; Alburaki et al. 2023), to help exemplify how geographic sampling and admixture may influence genetic differences in *vg*. See methods for specific details on the sampling protocol.

**FIGURE 2:**
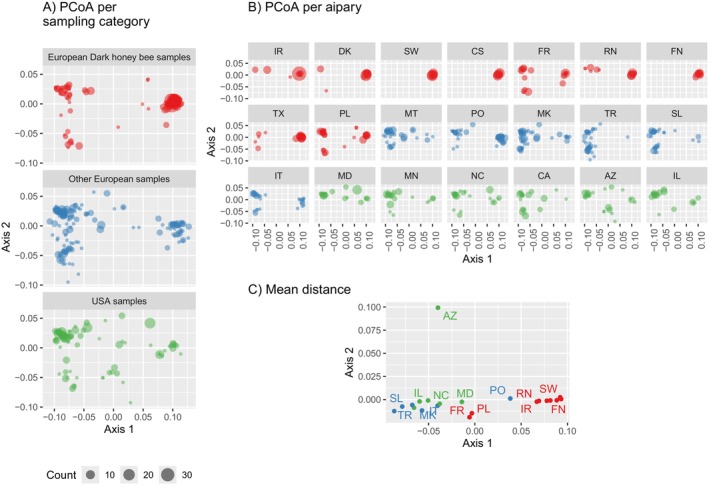
(A) Two‐dimensional PCoA of the all haplotypes identified in Figure S1, and the abundance of the haplotypes are shown with different‐sized spheres. Sampling categories: Dark European honey bee samples (red), the other European samples (blue), and the USA samples (green). (B) Same plot as in panel A, but split by apiary. The red *A.m.m* apiaries are IR: Ireland, DK: Denmark, SW: Sweden, CS: Colonsay in Scotland, FR: France, RN: Rena in Norway, FN: Flekkefjord in Norway, TX: Texel in the Netherlands and PL: Poland. The blue apiaries are MT: *A. m. ruttneri* from Malta, PO: *A. m. iberiensis* from Portugal, MK: *A. m. Macedonia* from North‐Macedonia, TR: *A. m. anatolica* from Turkey, SL: *A. m. carnica* from Slovenia and IT: *A. m. ligustica* from Italy. The green apiaries are MD: Maryland, MN: Minnesota, NC: North Carolina, CA: California, AZ: Arizona, and IL: Illinois. (C) Using the same PCoA plot as in panel A to show the mean distance between the apiaries. The colors and labels are consistent with panels A and B.

Visualizing the PCoA results by sampling categories revealed regional differences in haplogroup abundance (Figure 2A; Figure S5). The relative frequency of the two main haplogroups varies significantly among sampling categories (Chi‐square test of independence: x^2^ = 386, df = 2, *p* < 10^−84^): 86.5% of haplotypes belong to haplogroup 1 in European Dark honey bee samples, 24.3% in bees of other European origin, and 19.6% in bees sampled in the USA. Of haplotypes belonging to haplogroup 2, 13.5% were from European Dark honey bee samples, 75.7% from other European honey bees, and 80.4% from USA samples (Figure S6).

We also estimated the global *F*_ST_ by sampling category and found consistently low values (≤ 0.017, Figure S4). Removing recombinant haplotypes had little effect on *F*_ST_ in the European Dark honey bee samples and the USA samples, but slightly increased *F*_ST_ in the other European honey bee samples (0.015 vs. 0.020), which may reflect the high frequency of both common haplogroups in these samples (Figure S6). The stable *F*_ST_ values for the Dark European honey bee samples suggest that recombinant haplotypes have limited influence on genetic differentiation there. The very low *F*_ST_ in the USA samples, albeit with both haplogroups present (Figure S6), may reflect more homogenous allele frequencies among the USA samples (Table S2). Pairwise *F*_ST_ values are consistent with the structure seen in the PCoA: European Dark honey bee samples are more differentiated from other sampling categories (0.047–0.048), whereas the other European samples and the USA samples are similar (0.002, Figure S7A). Excluding recombinant haplotypes increased differentiation between the European Dark honey bee samples and the other categories (0.067–0.073) but had little effect on the difference between the other European and USA samples (Figure S7B). Taken together, these results indicate low overall genetic differentiation at the *vg* locus, and recombination has a minor impact on the *F*_ST_ estimates. The low *F*_ST_ likely reflects that both haplogroups are shared among sampling categories, although at different frequencies.

The presence of the two common haplogroups in our samples, along with their uneven distribution across the sampling categories, raises the question of how these haplogroups originated. Given that our samples include subspecies largely from the M and C lineages, this pattern could reflect broader lineage history at the *vg* locus rather than strong isolation among the sampling categories. In this scenario, the PCoA clustering may reflect divergence between lineage‐associated *vg* haplogroups, whereas the low *F*_ST_ values suggest that these haplogroups are shared across sampling categories, possibly due to historical or ongoing gene flow. However, distinguishing lineage history from locus‐specific processes would require genome‐wide ancestry information or additional neutral loci, which are not part of the present single‐locus dataset. Alternatively, the two haplogroups may have been formed in sympatry under balancing selection. To explore this latter hypothesis, we applied the method of Soni et al. (2022) in which the number of non‐synonymous and synonymous polymorphisms that are shared between sampling categories is compared to those that are private. We find no consistent signal of balancing selection, and in no case is there a significant excess of shared non‐synonymous polymorphisms that would be indicative of balancing selection (Figure S8). However, it is possible that balancing selection acts on variants linked to the *vg* gene. If this is the case, we might expect *F*_ST_ between the two haplogroups to increase or decrease along the length of the gene (Charlesworth 2006; Koenig et al. 2019), which we do not find support for in our data (Figure S9). In conclusion, our data are consistent with a model in which the haplogroups emerged independently within the M and C lineages, rather than being maintained primarily by balancing selection within the sampled categories.

Next, we further split our dataset into 21 geographically distinct apiaries (Figure 2B) to assess whether the patterns observed across the sampling categories were also present among individual apiaries. Each sampled apiary is named with a two‐letter code based on the country or state where each apiary is located (see Methods for a complete overview). We find both haplogroups in most apiaries, except for three European Dark honey bee apiaries with only haplogroup 1 (SW, CS, and FN). By calculating the average Hamming distances between the apiaries' haplotype sequences, we find that 7 of the 9 European Dark honey bee apiaries cluster together (Right in Figure 2C), while most of the other European and the USA apiaries cluster together (Left in Figure 2C). Interestingly, two European Dark honey bee apiaries (PL and FR) are closer to the other European and USA apiaries than the European Dark honey bee apiaries, which suggests that these apiaries have a higher frequency of the haplogroup 2 than haplogroup 1. We find the opposite for the *A. m. iberiensis* honey bee apiary (PO), which clusters with the European Dark honey bee apiaries rather than the other European apiaries, suggesting that PO has a high frequency of haplogroup 1. This finding is consistent with whole‐genome analysis, which shows that *A. m. iberiensis* is the closest related subspecies in Europe to the European Dark honey bees, and it is considered part of the M‐lineage (Han et al. 2012). Calculating the pairwise *F*_ST_ between the 21 geographically distinct apiaries shows the same trend: The European Dark honey bees apiaries are more genetically differentiated from the other apiaries, while there are few differences between the other European and the USA apiaries, both including (Figure S7C) and excluding recombinant haplotypes (Figure S7D). The pairwise *F*_ST_ between apiaries also highlights that the FR and PL apiaries are distinct European Dark honey bees apiaries, and the PO apiary is closer to European Dark honey bees apiaries than the other European apiaries, independent of recombination (Figure S7C,D).

Taken together, we also find low *F*_ST_ values among geographically distinct apiaries, suggesting weak overall differentiation at the *vg* locus. Together with the strong haplotype clustering, this indicates that the two divergent *vg* haplogroups are unevenly distributed among sampling categories rather than completely separated among apiaries. Our data show that the common *vg* haplogroup 1 is biased toward European Dark honey bee samples. There are several possible explanations for this pattern, including the evolutionary history of honey bee lineages, differences in the extent of mixed ancestry among the sampled apiaries, and restricted gene flow resulting from conservation management. A commonly proposed explanation is that the past glacial periods promoted geographic separation of honey bee populations in Europe. When the climate warmed, the honey bees spread across Europe and mixed (Hassett et al. 2018). In such a scenario, periods of geographic separation could have contributed to divergence at the *vg* gene, followed by population expansion and increased gene flow driving redistribution of haplogroups across both lines. This may help explain why both haplogroups are observed across many sampling locations, while at the same time, conserved samples with restricted gene flow show a high frequency of a single haplogroup. Given that our dataset contains only sequences from one gene, we do not attempt to reconstruct the population history; instead, we present this scenario as a plausible historical context for the observed *vg* haplogroup pattern.

### 2.4 Candidate Functional Divergence of the *vg* Haplogroups

**FIGURE 3:**
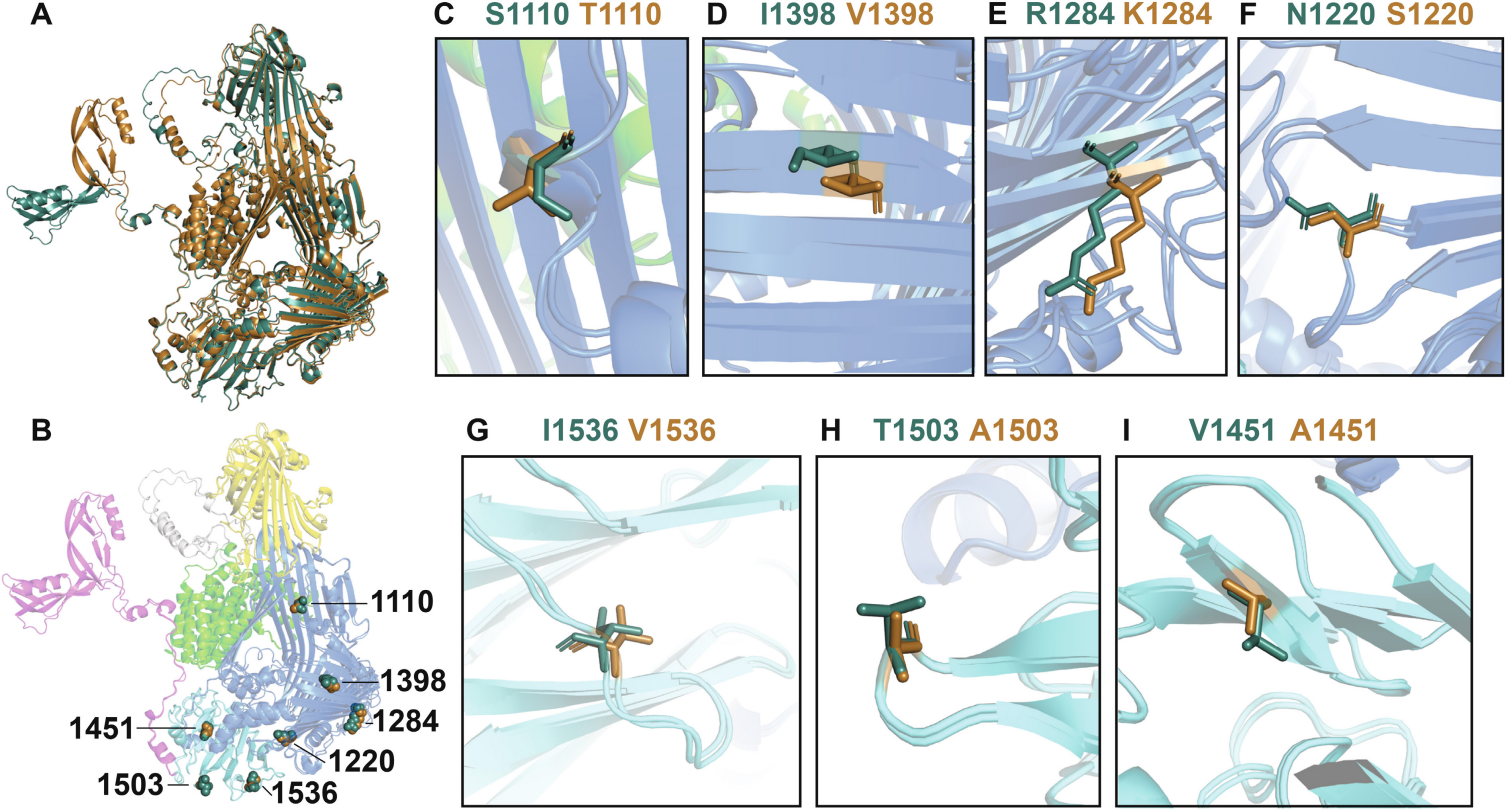
(A) Superimposition of the AlphaFold 3 predictions of Vg haplogroup 1 (teal) and Vg haplotype 2 (brown). (B) Vg colored by domains: β‐barrel (yellow), polyserine linker (gray), ɑ‐helical domain (green), lipid‐binding cavity (blue), the vWF domain (cyan), and the C‐terminal region (magenta). The nsSNPs are shown as spheres, colored teal in haplogroup 1 and brown in haplogroup 2. (C–I) Zooming in on the nsSNPs showing the amino acids at each position as sticks. Same coloring as in panel B.

Of the 65 common SNPs that distinguish the haplogroups (Table S1), only seven lead to amino acid changes (p.S1110T, p.N1220S, p.R1284K, p.I1398V, p.V1451A, p.T1503A, p.I1536V). These are all located in the lipid‐binding cavity (including the vWF domain) of the Vg. We used AlphaFold 3 to predict the structures of Vg, including the nsSNPs found in either haplogroup 1 or haplogroup 2 (Figure 3). The overall structures are similar (RMSD 0.541), and the predictions show no major structural differences at any of the seven sites. The seven changes are predicted to have a moderate impact on amino acid properties (the substituted amino acids are similar in hydrophobicity, size, and charge, see Table 3 in methods for calculations). The lipid‐binding cavity is a highly variable region in honey bee Vg (Leipart et al. 2022), and our predictions are consistent with broadly similar overall protein structures between the two haplogroups and with the lipid‐binding cavity being relatively tolerant of moderate amino‐acid substitutions. Despite this, the positioning of all differences within the lipid‐binding cavity is notable. The mechanism of lipid binding in Vg is poorly understood. Although hydrophobic forces primarily drive lipid binding (Anderson et al. 1998), there is a wide diversity of lipid molecules that bind to Vg (Redshaw and Follett 1971; Meininger et al. 1984; Beenakkers et al. 1985; Fremont and Riazi 1988), including phospholipids with different headgroup charges and variable hydrocarbon‐chain lengths. It has not been studied whether Vg has additional binding mechanisms beyond hydrophobic interactions or whether changes in these forces affect lipid binding. Accordingly, we cannot exclude the possibility that these nsSNPs influence binding affinity or specificity. The concentration of amino acids changes in a single functional region, highlighting a noteworthy distinction between the haplogroups. It is possible that these changes reflect lineage or population history, or weaker purifying constraints in this region, but they may also result from subtle adaptations to differences in lipid composition associated with diet or environment. Similar patterns have been suggested for *A. m. iberiensis* honey bees (Henriques, Wallberg, et al. 2018). While our findings are suggestive of the potential for local adaptation to have shaped lipid‐binding properties of Vg, more work is needed to explore that possibility.

It is also important to consider the 58 silent changes, which alter nucleotides in the introns and exons without altering the protein sequence. Such changes have been traditionally considered neutral for protein function and may have increased due to genetic hitchhiking. However, such genetic variations have also been demonstrated to alter mRNA stability, gene regulation, gene expression, and splicing (references within (Gazave et al. 2007; Hane et al. 2016; Bampi et al. 2020)). Also, modifying the expression of pleiotropic genes with titer‐dependent functions is suggested to be an efficient regulatory mechanism, as it avoids undesired effects on other processes (Kay et al. 2025). Further studies are needed to determine whether different *vg* haplogroups lead to regulatory differences. Such studies could test whether haplogroups differ, for example, in *vg* expression levels or transcription stability.

### 2.5 The *vg* Gene Shows Patterns Consistent With Positive Selection

The honey bee *vg* gene has previously been reported to show patterns consistent with positive selection (Kent et al. 2011), although prior analyses were limited to a small number of populations. Here, we use three different McDonald & Kreitman tests (iMKT webserver: standard MKT, Fay, Wyckoff, and Wu correction, and extended MKT (Murga‐Moreno et al. 2019)) to estimate the fractions of potentially adaptive nsSNPs, differing in how they handle slightly deleterious mutations. We combined polymorphism data across our *A. mellifera* samples to maximize power for single‐locus analyses and compared SNPs to divergence from *A. florea* . Across MK implementations, results were similar, and excluding predicted recombinant sequences had little effect (Figure S10), suggesting that the evidence for positive selection is not driven primarily by recombinant haplotypes. Positive selection could favor variants within functionally important regions, while recombination occurs elsewhere along the gene. However, because MK approaches compare SNP‐site patterns, these results should be interpreted as evidence for positive selection on nsSNPs within the *vg* locus, rather than as evidence that selection maintains a *vg* haplotype. Although the haplogroups contain a specific combination of nsSNPs that may have functional effects (Figure 3), our recombination and balancing‐selection analyses do not support strong selection maintaining these combinations as haplogroups.

Overall, all methods yielded results consistent with a contribution of positive selection at *vg*. We estimate that 67% of the nsSNPs at *vg* are adaptive (*p*‐value: 0.0001, Table 1 and Table S3). This estimate is in line with a higher inferred rate of adaptive nsSNPs (omegaA: 0.226) compared to non‐adaptive nsSNPs (omegaNA: 0.114). The extended MKT results are also consistent with substantial purifying selection, with most nsSNPs inferred to be strongly deleterious (d: 0.892). Taken together, we find evidence consistent with novel beneficial nsSNPs being relatively rare at *vg*, but when they arise, they may be quickly fixed (i.e., ⍺ estimates are high, Table 1), as supported by previous work showing that advantageous mutations contribute little to polymorphism and predominantly to divergence because they can fix quickly under positive selection (Murga‐Moreno et al. 2019; Soni et al. 2022; Castellano et al. 2025).

**TABLE 1:** Results from Extended MKT estimates. We report the fraction of nsSNPs estimated to be driven by positive selection, adaptive, non‐adaptive, neutral, strongly deleterious, and weakly deleterious in the dataset, both with and without recombinant haplotypes. Excluded recombinant (excl. recomb) haplotypes are reported in parentheses in columns 2 and 3.

Extended MKT estimates	Fractions (excl. recomb)	*p* (excl. recomb)
nsSNPs driven by positive selection (⍺)	0.665 (0.663)	0.0001 (0.0001)
Adaptive nsSNPs (omegaA)	0.226 (0.224)	
Non‐adaptive nsSNPs (omegaNA)	0.114 (0.113)	
strongly deleterious nsSNPs (d)	0.892 (0.890)	
weakly deleterious nsSNPs (b)	0.006 (0.003)	
neutral nsSNPs (f)	0.114 (0.113)	

### 2.6 Rare Changes Unique to Honey Bee Locations

Many of the variants contributing to differences among sampling locations were relatively rare (MAF < 0.25). Of these, 40 nsSNPs and 3 deletions were observed only in specific locations and/or apiaries (Tables S4 and S5). Most of these variants are very rare (found in 1–2 samples, MAF < 0.1), except for three nsSNPs and one deletion (0.25 > MAF > 0.1). The deletion shortens a loop region in the most conserved domain in honey bee Vg, the β‐barrel domain, and has been demonstrated to confer no structural changes to Vg (Leipart et al. 2025). The deletion is abundant in our European Dark honey bee samples. Two of the three nsSNPs (p.T1567M, p.M1559I) are also unique to specific European Dark honey bee apiaries. p.T1567M, p.M1559I are exposed inside the lipid binding cavity, and the amino acid change increases hydrophobicity. We also identified an alternative nsSNP at the 1567 position (p.T1567K), observed in two USA apiaries. In this case, the amino acid change decreases the hydrophobicity and introduces a positive charge. Although this nsSNP has a low MAF (0.07), we observed multiple amino acid changes at position 1567 with distinct physicochemical properties across sampling locations. This could indicate that this site tolerates a range of amino acid properties without major consequences for overall protein function. Alternatively, variation at this position could influence lipid‐binding specificity or affinity; for example, different amino acid properties might interact with lipids of different charge. The last location‐specific nsSNPs with an MAF > 0.1, p.I489V, is observed in the AZ apiary in the USA and is located at the surface of the α‐helical domain. This Vg domain is shown to interact with lipids at the surface of Gram‐negative and Gram‐positive bacteria (Havukainen et al. 2013). Although these three nsSNPs are not fixed at specific locations or apiaries, we suggest they are candidates for follow‐up work to test for potential functional effects. Such variants may help motivate hypotheses about ecological differences, such as pathogen exposure or diet, related to Vg variation across the sampled locations. Further studies could test whether these nsSNPs alter protein structure, stability, and ligand interactions.

## 3 Conclusion

This population‐genetic study of the honey bee *vg* gene shows that the gene has two major haplogroups, with one of them biased toward the European Dark honey bee conservatory samples, where gene flow is restricted. Several of these sampled locations have been reported to show evidence consistent with local adaptation to their climate and environment (Büchler et al. 2014; Meixner et al. 2015; Schaumann et al. 2024; Parejo et al. 2025). Although enrichment of a *vg* haplogroup in such samples is consistent with the possibility that *vg* contributes to environmentally relevant variation, lineage history, admixture, and management practices could also contribute to the observed patterns. Furthermore, the SNPs that differentiate the two haplogroups include amino acid changes concentrated in the lipid‐binding region as well as synonymous and intronic variants. Together, these findings motivate the hypothesis that lipid‐binding properties and/or gene regulation may differ among haplogroups, but additional functional and genomic‐context data are needed to evaluate these possibilities. Comparing *vg* to genome‐wide patterns of variation would help determine whether the observed level at *vg* is distinct from the genomic background and clarify whether *vg* has a unique evolutionary history. Finally, our results are consistent with a balance between strong constraints on much of the protein and signals compatible with selection at the gene, suggesting that some aspects of *vg* remain conserved due to essential roles, whereas others may be more evolutionarily flexible.

## 4 Material and Methods

### 4.1 Sampling and Dataset

This study uses a published dataset generated by our research group through targeted Nanopore sequencing (Leipart et al. 2022). This dataset contains phased haplotype consensus sequences. For each of 543 diploid *Apis mellifera* samples, raw reads were phased into the two *vg* alleles (haplotypes), and a separate consensus sequence was generated for each allele (see Leipart et al. 2022 for the bioinformatic pipeline). Sampling was designed to capture broad geographic and genetic variation for the *vg* gene. Samples were collected from 21 apiaries across Europe and the USA, including multiple subspecies and regions. European samples were obtained from breeding programs and conservatories to represent genetically characterized (largely purebred) subspecies and different geographic regions. The 9 protected *A. m. mellifera* apiaries were selected and sampled based on earlier introgression studies (Pinto et al. 2014; Henriques, Browne, et al. 2018). All samples from Europe were provided by researchers and managers of breeding associations working with each subspecies to ensure that samples were obtained from purebred populations. The USA samples were included to reflect geographically distributed populations with mixed ancestry, covering the north, west, south, northeast, east, and central regions. To ensure genetic variation among the samples, the collectors in Europe and the United States sampled 25–33 bees from three to six separate hives in their apiaries. This sample size was chosen to provide a 90% probability of detecting alleles with frequencies ≥ 5% (Hale et al. 2012). See Table 2 for information on the dataset's samples.

**TABLE 2:** The sample location is listed in column 1. The unique identifier for each apiary is listed in column 2 (ID), and the number of samples per apiary is listed in column 3 (N). The subspecies is listed in column 4. The collectors in Europe and the USA are acknowledged in column 5.

Location of apiary (Region/state, country)	ID	N	Subspecies (all *Apis mellifera* )	Acknowledgments
Flekkefjord, Norway	FN	27	*mellifera*	Anja Laupstad Vatland, Molti AS
Rena, Norway	RN	32	*mellifera*	Tor Erik Rødsdalen, Norsk brunbielag
Jämtland, Sweden	SW	30	*mellifera*	Ingvard Arvidsson, Nordbiföreningen
Læsø, Denmark	DK	31	*mellifera*	Flemming Vejsnæs, Danish Beekeepers Association
Isle of Colonsay, Scotland	CS	26	*mellifera*	Andrew Abrahams, Colonsay Black Bee Reserve
Connemara, Ireland	IR	27	*mellifera*	Gerard Coyne, The Native Irish Honey Bee Society
Augustów Primeval Forest, Poland	PL	28	*mellifera*	Małgorzata Bienkowska, Research Institute of Horticulture
Texel, the Netherlands	TX	29	*mellifera*	Romée van der Zee, Dutch Center for Bee Research
Les Belleville, France	FR	28	*mellifera*	Klébert Silvestre, Technical Apicultural Studies of Savoie
Lukovica, Slovenia	SL	25	*carnica*	Peter Kozmus, Breeding Program for Carniolan Honeybee for the Slovenian Beekeepers' Associations
Bolonga, Italy	IT	22	*ligustica*	Cecilia Costa, Council for Agriculture Research and Agricultural Economy Analysis
Bragança, Portugal	PO	28	*iberiensis*	Maria Alice de Silva Pinto, Instituto Politécnico de Bragança
Skopje, North‐Macedonia	MK	32	*macedonica*	Aleksandar Uzunov, Agricultural Sciences and Food
Valletta, Malta	MT	28	*ruttneri*	Thomas Galea, Malta Beekeepers Association
Ankara, Turkey	TR	21	*anatolica*	Irfan Kandemir, Ankara University
Minnesota, USA	MN	22	*mixed*	Adam G. Dolezal, University of Illinois
California, USA	CA	20	*mixed*	Randy Oliver, Scientific Beekeeping
Arizona, USA	AZ	20	*mixed*	Tim Kenney, Red Mountain Cattle Company
Maryland, USA	MD	22	*mixed*	Jay Evans, United States Department of Agriculture
North‐Carolina, USA	NC	22	*mixed*	Olav Rueppell, University of North Carolina at Greensboro
Illinois, USA	IL	22	*mixed*	Marla Spivak and Mike Goblirsch, University of Minnesota

Our study additionally uses the *A. m. mellifera* reference sequence (NCBI Gene ID: 406088) and orthologous sequences from *A. florea* (100870965), *A. dorsata* (102673109), *A. cerana* (108000069), and *A. laboriosa* (122712558). Our sequences, the reference sequence, and sequences from sister species were aligned using MAFFT v.7.490 (Katoh and Standley 2013) to generate a multiple‐sequence alignment (MSA). When aligning the sequences, we used default options for DNA alignment and did not use separate alignment methods for UTRs, exons, or introns. After alignment, we truncated the MSA to the boundaries of the coding region of the *A. mellifera* reference sequence. We also corrected gaps at this time that would affect exon phases, treating them as likely artifacts. Next, we created a phased variant call file (VCF) from the truncated MSA for the 543 diploid *A. mellifera* samples using the *A. mellifera* reference sequence. All positions were reported in this VCF, including those with single‐nucleotide variants, insertions, deletions, and no variants. Additionally, for each site, we estimated the ancestral allele for our samples using data from the sister species and estimated the minor allele frequency (MAF). MSA truncation and VCF creation was done using custom R scripts (v. 2023.12.0) (R Core Team 2021). See GitHub https://github.com/vleipart/ApisVg.

### 4.2 PCoA, MAF, and Identifications of Haplotypes

Using custom R scripts, we filtered our initial VCF to retain only biallelic sites in *A. mellifera* with an identified reference allele. Next, we converted the phased genotypes of the filtered sites into haplotype strings, assigning 0 to the ancestral allele and 1 to the derived allele (the full haplotypes). We then counted the frequency of each full haplotype and calculated the Hamming distance between all pairs of haplotypes. These distances were used to generate PCoA plots of the full haplotypes.

Next, we generated a histogram of MAF values and identified a break at around 0.25 (Figure 1A). To minimize the influence of potentially recent (and rare) variation, we used this observed break to distinguish common from lower‐frequency variation. Importantly, the PCoA structure was robust when lower‐frequency variants were excluded (Figure S2), and the variants MAF > 0.25 explain 70% of the variance. To investigate high‐frequency variants further, we selected 65 positions with MAF > 0.25 and constructed new haplotypes (the high‐MAF haplotypes) from these positions. Importantly, the two most common high‐MAF haplotypes did not share any derived alleles, suggesting that these haplotypes represent independent haplogroups. Therefore, we chose to use these two most common high‐MAF haplotypes as standard haplotypes for two separate haplogroups. We used a Hamming distance threshold of 0.2 to assign haplotypes to haplogroups, as this value aligns with the separation of the two main clusters observed in the PCoA (Figure 1D) and provides a consistent classification of haplotypes into the two groups. Haplotypes with Hamming distance 0–0.2 to one reference were assigned to haplogroup 1, and those with distance 0.8–1 to the other were assigned to haplogroup 2.

### 4.3 Identifying Recombination

We identified recombinant full haplotypes based on their corresponding high‐MAF haplotypes. A high‐MAF haplotype was considered recombinant if it contained fragments from both standard haplotypes of the two separate haplogroups. Because the standards contained a mixture of derived and ancestral alleles, we calculated the ∆_2_ statistic (Boni et al. 2007; Lam et al. 2018) for each high‐MAF haplotype to detect recombinant high‐MAF haplotypes. ∆_2_ describes how many mutations between a haplotype and its closest standard can be explained by allowing for one or two recombination events. A larger ∆_2_ provides more evidence that a haplotype is the product of recombination between the two standard haplogroups. After inspection of the ∆_2_ distributions and the positions of the haplotypes on the PCoA, we found that ∆_2_ ≥ 4 identified haplotypes clearly made up of segments from haplogroups 1 and 2 and therefore selected this as our threshold for identifying a sequence as “recombinant”.

The formula for calculating ∆_2_ is

$${∆}_{2}=d_{\text{NoRec}}-d_{\mathrm{Rec},2}$$

where d_NoRec_ is the minimum distance between a haplotype and the standard haplotypes, and d_Rec,2_ is the minimum distance between the haplotype and hypothetical 0‐, 1‐, or 2‐breakpoint recombinant sequences between the two standard haplotypes. We made a slight modification to the procedure and also included the inferred ancestral haplotype as part of the standard set, although we did not generate any hypothetical recombinants between the ancestor and the other standard haplotypes.

### 4.4 *F*_ST_ Analysis

The aligned MSA with the consensus sequence was used as input to the *F*_ST_ calculations. The calculations were done in the R package hierfstat (Goudet 2005). Global *F*_ST_ was calculated across all samples and for the three sampling categories (Figure S4). Pairwise *F*_ST_ was calculated for every pair of sampling categories (Figure S7A,B) and every pair of apiaries (Figure S7C,D). The R package pairwise. WCfst returns a matrix with values per pair, which are visualized in the heatmaps in Figure S7, and csv files are available on GitHub. All *F*_ST_ values were calculated both with and without putative recombinant haplotypes. Pairwise *F*_ST_ was also calculated between haplogroups 1 and 2 at every position along the gene. For this, we used the aligned MSA with the consensus sequences and filtered out any sequence with a hamming distance of 0.2 or higher, as we did to determine haplogroups before (see above). We labeled each sample with haplogroup (hg) 1 or 2. The results are shown in Figure S9.

### 4.5 Structural Modeling

We predicted the protein structures of Vg haplogroups 1 and 2 by introducing mutations on the Vg sequence (UniProt ID: Q868N5) using AlphaFold 3 (Abramson et al. 2024). We used the highest‐ranked prediction for each model. Overall, Vg haplogroups 1 and 2 are similar with a root‐mean‐square deviation of 0.541. We used various parameters to assess differences among the seven nsSNPs. We considered the Grantham distance, which estimates the evolutionary distance between two amino acids based on chemical composition, polarity, and molecular volume. The higher the distance, the more deleterious the substitution is expected to be (Grantham 1974). A distance < 100 is considered moderate. Previously, we categorized the mutations as moderate using a substitution matrix, where a positive score is considered moderate (Leipart et al. 2022). We also calculated the differences in the relative solvent accessible surface area (rASA), calculated in PyMol (Schrödinger, n.d.), which indicates how exposed the residue is at the specific position in the protein structure, and we considered < 20% as buried (Tien et al. 2013; Savojardo et al. 2021). We also calculated the differences in molecular mass (Da) and hydropathy index (Kyte and Doolittle 1982). The results are summarized in Table 3.

**TABLE 3:** The positions of seven nsSNPs are listed in column 1, and the amino acids found in haplogroup 1 and haplogroup 2 are listed in columns 2 and 3, respectively. Column 4–8 lists different parameters calculated for each amino acid change and the position in the structures: Grantham distance (GD), substitution matrix from Leipart et al. (2022) (SM), the difference in hydrophobicity index (HI difference), molecular mass (MM difference), and rASA.

Pos aa	Hg1	Hg2	GD	SM	HI difference	MM differences	rASA difference
1110	S	T	58	1	0.1	14.03	16
1220	N	S	46	1	2.7	27.03	4
1284	R	K	26	2	0.6	28.01	6
1398	I	V	29	3	0.3	14.03	3
1451	V	A	64	0	2.4	28.05	0
1503	T	A	58	0	2.5	30.03	0
1536	I	V	29	3	0.3	14.03	2

### 4.6 MKT Analysis

The aligned MSA with the consensus sequence, reference and outgroup *A. florea* were used as input to the iMKT webserver (Murga‐Moreno et al. 2019). We used the iMKT webserver to generate Derived Allele Frequency (DAF) and divergence files (Div). Standard MKT, Fay, Wyckoff, and Wu (FWW) correction and Extended MKT (referred to as DGPR at the iMKT website and R) were calculated using the R package iMKT (McDonald and Kreitman 1991; Fay et al. 2001; Mackay et al. 2012; Murga‐Moreno et al. 2019). The standard MK test makes no correction; the method of Fay et al. (2001) removes polymorphisms with minor allele frequency below 10%, and the extended MK test uses a more elaborate method to achieve the same aim (Mackay et al. 2012).

### 4.7 Soni et al. (2022) Analysis

The balancing selection was calculated based on the number of private shared nsSNPs and sSNPs (Figure S8). The Soni et al. (2022) test compares patterns of shared and private nsSNPs and sSNP. A *Z* > 1 indicates a higher‐than‐expected level of shared nsSNPs between the populations consistent with balancing selection on some of the nsSNPs (Soni et al. 2022).

All plots were generated in R using custom scripts (See GitHub https://github.com/vleipart/ApisVg). To analyze and identify unique variants and translate the consensus, reference, and outgroups sequences to protein sequences, we used JalView (v.2.11.5.0) (Waterhouse et al. 2009). These results are shown in Table S1.

## Author Contributions

**Vilde Leipart:** data curation (lead), formal analysis (lead), investigation (equal), methodology (equal), project administration (supporting), software (supporting), validation (lead), visualization (lead), writing – original draft (lead), writing – review and editing (lead). **Reed A. Cartwright:** data curation (supporting), formal analysis (supporting), investigation (supporting), methodology (supporting), software (supporting), validation (supporting), visualization (supporting), writing – review and editing (supporting). **Adam Eyre‐Walker:** data curation (supporting), formal analysis (supporting), investigation (supporting), methodology (supporting), software (supporting), validation (supporting), writing – review and editing (supporting). **Simen R. Sandve:** data curation (supporting), formal analysis (supporting), investigation (supporting), methodology (supporting), validation (supporting), writing – original draft (supporting), writing – review and editing (supporting). **Gro V. Amdam:** conceptualization (lead), formal analysis (supporting), funding acquisition (lead), investigation (supporting), project administration (supporting), resources (lead), supervision (supporting), validation (supporting), writing – review and editing (supporting).

## Funding

This work is funded by the Research Council of Norway (grant numbers 335244 and 350231).

## Conflicts of Interest

The authors declare no conflicts of interest.

## Supporting information


**Figure S1:** ece373845‐sup‐0001‐supinfo.pdf. *Vg* polymorphisms, haplotypes, PCoA and explained variance.
**Figure S2:** PCoA of the haplotypes based on common polymorphisms.
**Figure S3:** PCoA of all the haplotypes with putative recombinant sequences labeled.
**Figure S4:** Global *F*_ST_ calculations.
**Figure S5:** Frequencies of samples with common haplotypes and haplogroups.
**Figure S6:** Frequency of haplogroups per sampling category.
**Figure S7:** Pairwise *F*_ST_ matrices.
**Figure S8:** Soni et al. (2022) test, private and shared nsSNPs.
**Figure S9:** Pairwise *F*_ST_ distribution along *vg*.
**Figure S10:** MKT methods.
**Table S1:** Summary of 65 common polymorphisms.
**Table S2:** Unique number of haplotypes per sampling category and apiary.
**Table S3:** MKT table.
**Table S4:** Location unique nsSNPs summary.
**Table S5:** Location unique deletion summary.

## Data Availability

Raw sequence reads and haplotype data are available on GitHub https://github.com/vleipart/ApisVg, Zenodo https://doi.org/10.5281/zenodo.20140979 and ENA (accession code: PRJEB113063). The Figures S1–S10 and Tables S1–S5 are included in the submission. Benefit‐Sharing Statement: Benefits from this research accrue from the sharing of our data and results on public databases as described above.

## References

[ece373845-bib-0001] Abramson, J. , J. Adler , J. Dunger , et al. 2024. “Accurate Structure Prediction of Biomolecular Interactions With AlphaFold 3.” Nature 630, no. 8016: 493–500. 10.1038/s41586-024-07487-w. 38718835 PMC11168924

[ece373845-bib-0002] Alburaki, M. , S. Madella , J. Lopez , M. Bouga , Y. Chen , and D. van Engelsdorp . 2023. “Honey Bee Populations of the USA Display Restrictions in Their mtDNA Haplotype Diversity.” Frontiers in Genetics 13: 1092121. 10.3389/fgene.2022.1092121. 36685818 PMC9845583

[ece373845-bib-0003] Amdam, G. V. , K. Norberg , A. Hagen , and S. W. Omholt . 2003. “Social Exploitation of Vitellogenin.” Proceedings of the National Academy of Sciences of the United States of America 100, no. 4: 1799–1802. 10.1073/pnas.0333979100. 12566563 PMC149913

[ece373845-bib-0004] Amdam, G. V. , K. Norberg , S. W. Omholt , et al. 2005. “Higher Vitellogenin Concentrations in Honey Bee Workers May Be an Adaptation to Life in Temperate Climates.” Insectes Sociaux 52, no. 4: 316–319. 10.1007/s00040-005-0812-2.

[ece373845-bib-0005] Amdam, G. V. , Z. L. Simões , A. Hagen , et al. 2004. “Hormonal Control of the Yolk Precursor Vitellogenin Regulates Immune Function and Longevity in Honeybees.” Experimental Gerontology 39, no. 5: 767–773. 10.1016/j.exger.2004.02.010. 15130671

[ece373845-bib-0006] Anderson, T. A. , D. G. Levitt , and L. J. Banaszak . 1998. “The Structural Basis of Lipid Interactions in Lipovitellin, a Soluble Lipoprotein.” Structure 6, no. 7: 895–909. 9687371 10.1016/s0969-2126(98)00091-4

[ece373845-bib-0007] Bampi, G. B. , A. S. Ramalho , L. A. Santos , et al. 2020. “The Effect of Synonymous Single‐Nucleotide Polymorphisms on an Atypical Cystic Fibrosis Clinical Presentation.” Life 11, no. 1: 14. 10.3390/life11010014. 33375403 PMC7824434

[ece373845-bib-0008] Beenakkers, A. M. , D. J. Van der Horst , and W. J. Van Marrewijk . 1985. “Insect Lipids and Lipoproteins, and Their Role in Physiological Processes.” Progress in Lipid Research 24, no. 1: 19–67. 10.1016/0163-7827(85)90007-4. 3916237

[ece373845-bib-0009] Bergmiller, T. , M. Ackermann , and O. K. Silander . 2012. “Patterns of Evolutionary Conservation of Essential Genes Correlate With Their Compensability.” PLoS Genetics 8, no. 6: e1002803. 10.1371/journal.pgen.1002803. 22761596 PMC3386227

[ece373845-bib-0010] Bertrand, B. , M. Alburaki , H. Legout , S. Moulin , F. Mougel , and L. Garnery . 2015. “MtDNA COI‐COII Marker and Drone Congregation Area: An Efficient Method to Establish and Monitor Honeybee ( *Apis mellifera* L.) Conservation Centres.” Molecular Ecology Resources 15, no. 3: 673–683. 10.1111/1755-0998.12339. 25335970

[ece373845-bib-0011] Beye, M. , I. Gattermeier , M. Hasselmann , et al. 2006. “Exceptionally High Levels of Recombination Across the Honey Bee Genome.” Genome Research 16, no. 11: 1339–1344. 10.1101/gr.5680406. 17065604 PMC1626635

[ece373845-bib-0012] Boni, M. F. , D. Posada , and M. W. Feldman . 2007. “An Exact Nonparametric Method for Inferring Mosaic Structure in Sequence Triplets.” Genetics 176, no. 2: 1035–1047. 10.1534/genetics.106.068874. 17409078 PMC1894573

[ece373845-bib-0013] Brosi, B. J. , K. S. Delaplane , M. Boots , and J. C. de Roode . 2017. “Ecological and Evolutionary Approaches to Managing Honeybee Disease.” Nature Ecology & Evolution 1, no. 9: 1250–1262. 10.1038/s41559-017-0246-z. 29046562 PMC5749923

[ece373845-bib-0014] Büchler, R. , C. Costa , F. Hatjina , et al. 2014. “The Influence of Genetic Origin and Its Interaction With Environmental Effects on the Survival of *Apis mellifera* L. Colonies in Europe.” Journal of Apicultural Research & Bee World 53, no. 2: 205–214. 10.3896/IBRA.1.53.2.03.

[ece373845-bib-0015] Carpenter, M. H. , and B. A. Harpur . 2021. “Genetic Past, Present, and Future of the Honey Bee ( *Apis mellifera* ) in The United States of America.” Apidologie 52, no. 1: 63–79. 10.1007/s13592-020-00836-4.

[ece373845-bib-0016] Castellano, D. , I. T. Vourlaki , R. N. Gutenkunst , and S. E. Ramos‐Onsins . 2025. “Detection of Domestication Signals Through the Analysis of the Full Distribution of Fitness Effects.” Peer Community Journal 5: e35. 10.24072/pcjournal.540. 40256351 PMC12007895

[ece373845-bib-0017] Charlesworth, D. 2006. “Balancing Selection and Its Effects on Sequences in Nearby Genome Regions.” PLoS Genetics 2, no. 4: e64. 10.1371/journal.pgen.0020064. 16683038 PMC1449905

[ece373845-bib-0018] Chávez‐Galarza, J. , D. Henriques , J. S. Johnston , et al. 2013. “Signatures of Selection in the Iberian Honey Bee ( *Apis mellifera iberiensis* ) Revealed by a Genome Scan Analysis of Single Nucleotide Polymorphisms.” Molecular Ecology 22, no. 23: 5890–5907. 10.1111/mec.12537. 24118235

[ece373845-bib-0019] Chen, C. , Z. Liu , Q. Pan , et al. 2016. “Genomic Analyses Reveal Demographic History and Temperate Adaptation of the Newly Discovered Honey Bee Subspecies *Apis mellifera* Sinisxinyuan n. Ssp.” Molecular Biology and Evolution 33, no. 5: 1337–1348. 10.1093/molbev/msw017. 26823447 PMC4839221

[ece373845-bib-0020] Cornelissen, B. , P. Neumann , and O. Schweiger . 2019. “Global Warming Promotes Biological Invasion of a Honey Bee Pest.” Global Change Biology 25, no. 11: 3642–3655. 10.1111/gcb.14791. 31394018 PMC6856679

[ece373845-bib-0021] De la Rúa, P. , R. Jaffé , R. Dall’Olio , I. Muñoz , and J. Serrano . 2009. “Biodiversity, Conservation and Current Threats to European Honeybees.” Apidologie 40, no. 3: 263–284. 10.1051/apido/2009027.

[ece373845-bib-0022] DeGrandi‐Hoffman, G. , and Y. Chen . 2015. “Nutrition, Immunity and Viral Infections in Honey Bees.” Current Opinion in Insect Science 10: 170–176. 10.1016/j.cois.2015.05.007. 29588005

[ece373845-bib-0023] Diaz, F. , C. W. Allan , and L. M. Matzkin . 2018. “Positive Selection at Sites of Chemosensory Genes Is Associated With the Recent Divergence and Local Ecological Adaptation in Cactophilic *Drosophila* .” BMC Evolutionary Biology 18, no. 1: 144. 10.1186/s12862-018-1250-x. 30236055 PMC6148956

[ece373845-bib-0024] Dogantzis, K. A. , T. Tiwari , I. M. Conflitti , et al. 2021. “Thrice Out of Asia and the Adaptive Radiation of the Western Honey Bee.” Science Advances 7, no. 49: eabj2151. 10.1126/sciadv.abj2151. 34860547 PMC8641936

[ece373845-bib-0025] Dogantzis, K. A. , and A. Zayed . 2019. “Recent Advances in Population and Quantitative Genomics of Honey Bees.” Current Opinion in Insect Science 31: 93–98. 10.1016/j.cois.2018.11.010. 31109680

[ece373845-bib-0026] Döke, M. A. , M. Frazier , and C. M. Grozinger . 2015. “Overwintering Honey Bees: Biology and Management.” Current Opinion in Insect Science 10: 185–193. 10.1016/j.cois.2015.05.014. 29588007

[ece373845-bib-0027] Dolezal, A. G. , J. Carrillo‐Tripp , T. M. Judd , W. Allen Miller , B. C. Bonning , and A. L. Toth . 2019. “Interacting Stressors Matter: Diet Quality and Virus Infection in Honeybee Health.” Royal Society Open Science 6, no. 2: 181803. 10.1098/rsos.181803. 30891288 PMC6408407

[ece373845-bib-0028] Dolezal, A. G. , and A. L. Toth . 2018. “Feedbacks Between Nutrition and Disease in Honey Bee Health.” Current Opinion in Insect Science 26: 114–119. 10.1016/j.cois.2018.02.006. 29764650

[ece373845-bib-0029] Doublet, V. , Y. Poeschl , A. Gogol‐Döring , et al. 2017. “Unity in Defence: Honeybee Workers Exhibit Conserved Molecular Responses to Diverse Pathogens.” BMC Genomics 18, no. 1: 207. 10.1186/s12864-017-3597-6. 28249569 PMC5333379

[ece373845-bib-0030] Dumont, B. L. 2020. “Evolution: Is Recombination Rate Variation Adaptive?” Current Biology 30, no. 8: R351–R353. 10.1016/j.cub.2020.02.061. 32315634

[ece373845-bib-0031] Eimanifar, A. , S. A. Brooks , T. Bustamante , and J. D. Ellis . 2018. “Population Genomics and Morphometric Assignment of Western Honey Bees ( *Apis mellifera* L.) in the Republic of South Africa.” BMC Genomics 19, no. 1: 615. 10.1186/s12864-018-4998-x. 30111292 PMC6094452

[ece373845-bib-0032] Engel, M. S. 1999. “The Taxonomy of Recent and Fossil Honey Bees (Hymenoptera: Apidea, *Apis*).” Journal of Hymenoptera Research 8, no. 2: 165–196.

[ece373845-bib-0033] Fay, J. C. , G. J. Wyckoff , and C. I. Wu . 2001. “Positive and Negative Selection on the Human Genome.” Genetics 158, no. 3: 1227–1234. 10.1093/genetics/158.3.1227. 11454770 PMC1461725

[ece373845-bib-0034] Fremont, L. , and A. Riazi . 1988. “Biochemical Analysis of Vitellogenin From Rainbow Trout (*Salmo gairdneri*): Fatty Acid Composition of Phospholipids.” Reproduction, Nutrition, Developpement 28, no. 4A: 939–952. 10.1051/rnd:19880607. 3244898

[ece373845-bib-0035] Gazave, E. , T. Marqués‐Bonet , O. Fernando , B. Charlesworth , and A. Navarro . 2007. “Patterns and Rates of Intron Divergence Between Humans and Chimpanzees.” Genome Biology 8, no. 2: R21. 10.1186/gb-2007-8-2-r21. 17309804 PMC1852421

[ece373845-bib-0036] Goudet, J. 2005. “Hierfstat, a Package for r to Compute and Test Hierarchical F‐Statistics.” Molecular Ecology Notes 5, no. 1: 184–186. 10.1111/j.1471-8286.2004.00828.x.

[ece373845-bib-0037] Grantham, R. 1974. “Amino Acid Difference Formula to Help Explain Protein Evolution.” Science 185, no. 4154: 862–864. 10.1126/science.185.4154.862. 4843792

[ece373845-bib-0038] Hale, M. L. , T. M. Burg , and T. E. Steeves . 2012. “Sampling for Microsatellite‐Based Population Genetic Studies: 25 to 30 Individuals Per Population Is Enough to Accurately Estimate Allele Frequencies.” PLoS One 7, no. 9: e45170. 10.1371/journal.pone.0045170. 22984627 PMC3440332

[ece373845-bib-0039] Han, F. , A. Wallberg , and M. T. Webster . 2012. “From Where Did the Western Honeybee ( *Apis mellifera* ) Originate?” Ecology and Evolution 2, no. 8: 1949–1957. 10.1002/ece3.312. 22957195 PMC3433997

[ece373845-bib-0040] Hane, M. , K. Kitajima , and C. Sato . 2016. “Effects of Intronic Single Nucleotide Polymorphisms (iSNPs) of a Polysialyltransferase, ST8SIA2 Gene Found in Psychiatric Disorders on Its Gene Products.” Biochemical and Biophysical Research Communications 478, no. 3: 1123–1129. 10.1016/j.bbrc.2016.08.079. 27565727

[ece373845-bib-0041] Harpur, B. A. , C. F. Kent , D. Molodtsova , et al. 2014. “Population Genomics of the Honey Bee Reveals Strong Signatures of Positive Selection on Worker Traits.” Proceedings of the National Academy of Sciences of the United States of America 111, no. 7: 2614–2619. 10.1073/pnas.1315506111. 24488971 PMC3932857

[ece373845-bib-0042] Hartl, D. L. , A. G. Clark , and A. G. Clark . 1997. Principles of Population Genetics. Sinauer associates.

[ece373845-bib-0043] Harwood, G. , V. Leipart , C. Elsik , J. C. F. Ng , F. Drabløs , and G. V. Amdam . 2025. “Evidence for Vitellogenin DNA‐Binding in Honey Bees.” Protein Science : A Publication of the Protein Society 34, no. 10: e70291. 10.1002/pro.70291. 40944436 PMC12432429

[ece373845-bib-0044] Hassett, J. , K. A. Browne , G. P. McCormack , et al. 2018. “A Significant Pure Population of the Dark European Honey Bee (* Apis mellifera mellifera*) Remains in Ireland.” Journal of Apicultural Research 57, no. 3: 337–350. 10.1080/00218839.2018.1433949.

[ece373845-bib-0045] Havukainen, H. , O. Halskau , and G. V. Amdam . 2011. “Social Pleiotropy and the Molecular Evolution of Honey Bee Vitellogenin.” Molecular Ecology 20, no. 24: 5111–5113. 22250301 10.1111/j.1365-294x.2011.05351.x

[ece373845-bib-0046] Havukainen, H. , D. Münch , A. Baumann , et al. 2013. “Vitellogenin Recognizes Cell Damage Through Membrane Binding and Shields Living Cells From Reactive Oxygen Species.” Journal of Biological Chemistry 288, no. 39: 28369–28381. 10.1074/jbc.M113.465021. 23897804 PMC3784755

[ece373845-bib-0047] Henriques, D. , K. A. Browne , M. W. Barnett , et al. 2018. “High Sample Throughput Genotyping for Estimating C‐Lineage Introgression in the Dark Honeybee: An Accurate and Cost‐Effective SNP‐Based Tool.” Scientific Reports 8, no. 1: 8552. 10.1038/s41598-018-26932-1. 29867207 PMC5986779

[ece373845-bib-0048] Henriques, D. , A. R. Lopes , N. Chejanovsky , et al. 2021. “A SNP Assay for Assessing Diversity in Immune Genes in the Honey Bee ( *Apis mellifera* L.).” Scientific Reports 11, no. 1: 15317. 10.1038/s41598-021-94833-x. 34321557 PMC8319136

[ece373845-bib-0049] Henriques, D. , A. Wallberg , J. Chávez‐Galarza , J. S. Johnston , M. T. Webster , and M. A. Pinto . 2018. “Whole Genome SNP‐Associated Signatures of Local Adaptation in Honeybees of the Iberian Peninsula.” Scientific Reports 8, no. 1: 11145. 10.1038/s41598-018-29469-5. 30042407 PMC6057950

[ece373845-bib-0050] Hilbert, Z. A. , P. E. Haffener , H. J. Young , M. J. W. Schwiesow , E. M. Leffler , and N. C. Elde . 2023. “Rapid Evolution of Glycan Recognition Receptors Reveals an Axis of Host–Microbe Arms Races Beyond Canonical Protein–Protein Interfaces.” Genome Biology and Evolution 15, no. 7: evad119. 10.1093/gbe/evad119. 37390614 PMC10329266

[ece373845-bib-0051] Ilyasov, R. A. , M. L. Lee , J. I. Takahashi , H. W. Kwon , and A. G. Nikolenko . 2020. “A Revision of Subspecies Structure of Western Honey Bee *Apis mellifera* .” Saudi Journal of Biological Sciences 27, no. 12: 3615–3621. 10.1016/j.sjbs.2020.08.001. 33304172 PMC7714978

[ece373845-bib-0052] Ilyasov, R. A. , A. V. Poskryakov , and A. G. Nikolenko . 2015. “New SNP Markers of the Honeybee Vitellogenin Gene (Vg) Used for Identification of Subspecies * Apis mellifera mellifera* L.” Genetika 51, no. 2: 194–199. 25966585

[ece373845-bib-0053] Insolia, L. , R. Molinari , S. R. Rogers , G. R. Williams , F. Chiaromonte , and M. Calovi . 2022. “Honey Bee Colony Loss Linked to Parasites, Pesticides and Extreme Weather Across the United States.” Scientific Reports 12, no. 1: 20787. 10.1038/s41598-022-24946-4. 36456591 PMC9714769

[ece373845-bib-0054] Katoh, K. , and D. M. Standley . 2013. “MAFFT Multiple Sequence Alignment Software Version 7: Improvements in Performance and Usability.” Molecular Biology and Evolution 30, no. 4: 772–780. 10.1093/molbev/mst010. 23329690 PMC3603318

[ece373845-bib-0055] Kay, T. , P. K. Piekarski , and D. J. C. Kronauer . 2025. “Convergent Evolution of a Conserved Molecular Network Underlies Parenting and Sociality.” Nature Reviews Genetics 27: 306–322. 10.1038/s41576-025-00903-5. 41188556

[ece373845-bib-0056] Kent, C. F. , C. F. Kent , A. Issa , A. C. Bunting , and A. Zayed . 2011. “Adaptive Evolution of a Key Gene Affecting Queen and Worker Traits in the Honey Bee, *Apis mellifera* .” Molecular Ecology 20, no. 24: 5226–5235. 10.1111/j.1365-294X.2011.05299.x. 21981322

[ece373845-bib-0057] Kent, C. F. , S. Minaei , B. A. Harpur , and A. Zayed . 2012. “Recombination Is Associated With the Evolution of Genome Structure and Worker Behavior in Honey Bees.” Proceedings of the National Academy of Sciences 109, no. 44: 18012–18017. 10.1073/pnas.1208094109. PMC3497793 23071321

[ece373845-bib-0058] Koenig, D. , J. Hagmann , R. Li , et al. 2019. “Long‐Term Balancing Selection Drives Evolution of Immunity Genes in Capsella.” eLife 8: e43606. 10.7554/eLife.43606. 30806624 PMC6426441

[ece373845-bib-0059] Kyte, J. , and R. F. Doolittle . 1982. “A Simple Method for Displaying the Hydropathic Character of a Protein.” Journal of Molecular Biology 157, no. 1: 105–132. 10.1016/0022-2836(82)90515-0. 7108955

[ece373845-bib-0060] Lam, H. M. , O. Ratmann , and M. F. Boni . 2018. “Improved Algorithmic Complexity for the 3SEQ Recombination Detection Algorithm.” Molecular Biology and Evolution 35, no. 1: 247–251. 10.1093/molbev/msx263. 29029186 PMC5850291

[ece373845-bib-0061] Leipart, V. , O. G. Carmona , C. Orengo , F. Fraternali , and G. V. Amdam . 2025. “Assessing Structure‐Function Impacts on Vitellogenin by Leveraging Allelic Variant Occurring in Honey Bee Subspecies * Apis mellifera mellifera* .” iScience 28: 113241. 10.1016/j.isci.2025.113241. 41054523 PMC12496180

[ece373845-bib-0062] Leipart, V. , J. Ludvigsen , M. Kent , et al. 2022. “Identification of 121 Variants of Honey Bee Vitellogenin Protein Sequences With Structural Differences at Functional Sites.” Protein Science 31, no. 7: e4369. 10.1002/pro.4369. 35762708 PMC9207902

[ece373845-bib-0063] Leroy, T. , P. Faux , B. Basso , S. Eynard , D. Wragg , and A. Vignal . 2024. “Inferring Long‐Term and Short‐Term Determinants of Genetic Diversity in Honey Bees: Beekeeping Impact and Conservation Strategies.” Molecular Biology and Evolution 41, no. 12: msae249. 10.1093/molbev/msae249. 39692632 PMC11653568

[ece373845-bib-0064] Luo, H. , F. Gao , and Y. Lin . 2015. “Evolutionary Conservation Analysis Between the Essential and Nonessential Genes in Bacterial Genomes.” Scientific Reports 5, no. 1: 13210. 10.1038/srep13210. 26272053 PMC4536490

[ece373845-bib-0065] Mackay, T. F. C. , S. Richards , E. A. Stone , et al. 2012. “The *Drosophila melanogaster* Genetic Reference Panel.” Nature 482, no. 7384: 173–178. 10.1038/nature10811. 22318601 PMC3683990

[ece373845-bib-0066] McDonald, J. H. , and M. Kreitman . 1991. “Adaptive Protein Evolution at the Adh Locus in *Drosophila* .” Nature 351, no. 6328: 652–654. 10.1038/351652a0. 1904993

[ece373845-bib-0067] Meininger, T. , R. Raag , S. Roderick , and L. J. Banaszak . 1984. “Preparation of Single Crystals of a Yolk Lipoprotein.” Journal of Molecular Biology 179, no. 4: 759–764. 10.1016/0022-2836(84)90167-0. 6502715

[ece373845-bib-0068] Meixner, M. D. , P. Kryger , and C. Costa . 2015. “Effects of Genotype, Environment, and Their Interactions on Honey Bee Health in Europe.” Current Opinion in Insect Science 10: 177–184. 10.1016/j.cois.2015.05.010. 29588006

[ece373845-bib-0069] Minaud, É. , F. Rebaudo , P. Davidson , et al. 2024. “How Stressors Disrupt Honey Bee Biological Traits and Overwintering Mechanisms.” Heliyon 10, no. 14: e34390. 10.1016/j.heliyon.2024.e34390. 39108870 PMC11301357

[ece373845-bib-0070] Morandin, C. , H. Havukainen , J. Kulmuni , K. Dhaygude , K. Trontti , and H. Helanterä . 2014. “Not Only for Egg Yolk—Functional and Evolutionary Insights From Expression, Selection, and Structural Analyses of Formica Ant Vitellogenins.” Molecular Biology and Evolution 31, no. 8: 2181–2193. 10.1093/molbev/msu171. 24895411

[ece373845-bib-0071] Munoz, I. , D. Henriques , L. Jara , et al. 2017. “SNPs Selected by Information Content Outperform Randomly Selected Microsatellite Loci for Delineating Genetic Identification and Introgression in the Endangered Dark European Honeybee (* Apis mellifera mellifera*).” Molecular Ecology Resources 17, no. 4: 783–795. 10.1111/1755-0998.12637. 27863055

[ece373845-bib-0072] Murga‐Moreno, J. , M. Coronado‐Zamora , S. Hervas , S. Casillas , and A. Barbadilla . 2019. “iMKT: The Integrative McDonald and Kreitman Test.” Nucleic Acids Research 47, no. W1: W283–W288. 10.1093/nar/gkz372. 31081014 PMC6602517

[ece373845-bib-0073] Nandakumar, M. , M. Lundberg , F. Carlsson , and L. Råberg . 2025. “Positive Selection on Mammalian Immune Genes—Effects of Gene Function and Selective Constraint.” Molecular Biology and Evolution 42, no. 1: msaf016. 10.1093/molbev/msaf016. 39834162 PMC11783303

[ece373845-bib-0074] NordGen . 2019. Second Plan of Action for the Conservation of the Nordic Brown Bee. NordGen Publication Series. Accessed: November 24, 2025. https://urn.kb.se/resolve?urn=urn:nbn:se:norden:org:diva‐5627.

[ece373845-bib-0075] Parejo, M. , P. Kryger , M. Bouga , R. Dall’Olio , I. Kandemir , and C. Costa . 2025. “Conserving Locally Adapted Honey Bees.” In Sustainable Honey Bee Breeding: A Scientific Guide for Future Beekeeping, edited by C. Costa , M. Meixner , N. Carreck , et al., 53–65. Springer Nature Switzerland. 10.1007/978-3-031-94204-4_4.

[ece373845-bib-0076] Parejo, M. , D. Wragg , L. Gauthier , A. Vignal , P. Neumann , and M. Neuditschko . 2016. “Using Whole‐Genome Sequence Information to Foster Conservation Efforts for the European Dark Honey Bee, * Apis mellifera mellifera* .” Frontiers in Ecology and Evolution 4: 140. 10.3389/fevo.2016.00140.

[ece373845-bib-0077] Perez, M. F. , and B. Lehner . 2019. “Vitellogenins ‐ Yolk Gene Function and Regulation in *Caenorhabditis elegans* .” Frontiers in Physiology 10, no. 1067: 1067. 10.3389/fphys.2019.01067. 31551797 PMC6736625

[ece373845-bib-0078] Pinto, M. A. , D. Henriques , J. Chávez‐Galarza , et al. 2014. “Genetic Integrity of the Dark European Honey Bee (* Apis mellifera mellifera*) From Protected Populations: A Genome‐Wide Assessment Using SNPs and mtDNA Sequence Data.” Journal of Apicultural Research 53, no. 2: 269–278. 10.3896/IBRA.1.53.2.08.

[ece373845-bib-0079] R Core Team . 2021. R: A Language and Environment for Statistical Computing. R Foundation for Statistical Computing. https://www.R‐project.org/.

[ece373845-bib-0080] Redshaw, M. R. , and B. K. Follett . 1971. “The Crystalline Yolk‐Platelet Proteins and Their Soluble Plasma Precursor in an Amphibian, *Xenopus laevis* .” Biochemical Journal 124, no. 4: 759–766. 10.1042/bj1240759. 5131731 PMC1177252

[ece373845-bib-0081] Ruttner, F. 1988. Biogeography and Taxonomy of Honeybees. Springer. 10.1007/978-3-642-72649-1.

[ece373845-bib-0082] Salmela, H. , G. V. Amdam , and D. Freitak . 2015. “Transfer of Immunity From Mother to Offspring Is Mediated via Egg‐Yolk Protein Vitellogenin.” PLoS Pathogens 11, no. 7: e1005015. 10.1371/journal.ppat.1005015. 26230630 PMC4521805

[ece373845-bib-0083] Salmela, H. , G. P. Harwood , D. Münch , et al. 2022. “Nuclear Translocation of Vitellogenin in the Honey Bee ( *Apis mellifera* ).” Apidologie 53, no. 1: 13. 10.1007/s13592-022-00914-9. 35309709 PMC8924143

[ece373845-bib-0084] Samuk, K. , B. Manzano‐Winkler , K. R. Ritz , and M. A. F. Noor . 2020. “Natural Selection Shapes Variation in Genome‐Wide Recombination Rate in *Drosophila pseudoobscura* .” Current Biology 30, no. 8: 1517–1528.e6. 10.1016/j.cub.2020.03.053. 32275873

[ece373845-bib-0085] Sarioğlu‐Bozkurt, A. , E. Topal , N. Güneş , et al. 2022. “Changes in Vitellogenin (Vg) and Stress Protein (HSP 70) in Honey Bee ( *Apis mellifera anatoliaca* ) Groups Under Different Diets Linked With Physico‐Chemical, Antioxidant and Fatty and Amino Acid Profiles.” Insects 13, no. 11: 985. 10.3390/insects13110985. 36354809 PMC9698881

[ece373845-bib-0086] Savojardo, C. , M. Manfredi , P. L. Martelli , and R. Casadio . 2021. “Solvent Accessibility of Residues Undergoing Pathogenic Variations in Humans: From Protein Structures to Protein Sequences.” Frontiers in Molecular Biosciences 7, no. 460: 626363. 10.3389/fmolb.2020.626363. 33490109 PMC7817970

[ece373845-bib-0087] Schaumann, F. , N. Norrström , M. Niklasson , and S. Leidenberger . 2024. “Ecological Comparison of Native (* Apis mellifera mellifera*) and Hybrid (Buckfast) Honeybee Drones in Southwestern Sweden Indicates Local Adaptation.” PLoS One 19, no. 8: e0308831. 10.1371/journal.pone.0308831. 39137198 PMC11321565

[ece373845-bib-0088] Scheffler, K. , D. P. Martin , and C. Seoighe . 2006. “Robust Inference of Positive Selection From Recombining Coding Sequences.” Bioinformatics 22, no. 20: 2493–2499. 10.1093/bioinformatics/btl427. 16895925

[ece373845-bib-0089] Schierup, M. H. , and J. Hein . 2000. “Consequences of Recombination on Traditional Phylogenetic Analysis.” Genetics 156, no. 2: 879–891. 10.1093/genetics/156.2.879. 11014833 PMC1461297

[ece373845-bib-0090] Schilcher, F. , and R. Scheiner . 2023. “New Insight Into Molecular Mechanisms Underlying Division of Labor in Honeybees.” Current Opinion in Insect Science 59: 101080. 10.1016/j.cois.2023.101080. 37391163

[ece373845-bib-0091] Schrödinger, L. n.d. “The PyMOL Molecular Graphics System, Version 3.0.”

[ece373845-bib-0092] Seehuus, S. C. , K. Norberg , U. Gimsa , T. Krekling , and G. V. Amdam . 2006. “Reproductive Protein Protects Functionally Sterile Honey Bee Workers From Oxidative Stress.” Proceedings of the National Academy of Sciences of the United States of America 103, no. 4: 962–967. 10.1073/pnas.0502681103. 16418279 PMC1347965

[ece373845-bib-0093] Solignac, M. , F. Mougel , D. Vautrin , M. Monnerot , and J. M. Cornuet . 2007. “A Third‐Generation Microsatellite‐Based Linkage Map of the Honey Bee, *Apis mellifera*, and Its Comparison With the Sequence‐Based Physical Map.” Genome Biology 8, no. 4: R66. 10.1186/gb-2007-8-4-r66. 17459148 PMC1896015

[ece373845-bib-0094] Soni, V. , M. Vos , and A. Eyre‐Walker . 2022. “A New Test Suggests Hundreds of Amino Acid Polymorphisms in Humans Are Subject to Balancing Selection.” PLoS Biology 20, no. 6: e3001645. 10.1371/journal.pbio.3001645. 35653351 PMC9162324

[ece373845-bib-0095] Sun, C. , L. Hu , S. Liu , Z. Gao , and S. Zhang . 2013. “Functional Analysis of Domain of Unknown Function (DUF) 1943, DUF1944 and von Willebrand Factor Type D Domain (VWD) in vitellogenin2 in Zebrafish.” Developmental and Comparative Immunology 41, no. 4: 469–476. 10.1016/j.dci.2013.07.005. 23867754

[ece373845-bib-0096] Switanek, M. , K. Crailsheim , H. Truhetz , and R. Brodschneider . 2017. “Modelling Seasonal Effects of Temperature and Precipitation on Honey Bee Winter Mortality in a Temperate Climate.” Science of the Total Environment 579: 1581–1587. 10.1016/j.scitotenv.2016.11.178. 27916302

[ece373845-bib-0097] Tien, M. Z. , A. G. Meyer , D. K. Sydykova , S. J. Spielman , and C. O. Wilke . 2013. “Maximum Allowed Solvent Accessibilites of Residues in Proteins.” PLoS One 8, no. 11: e80635. 10.1371/journal.pone.0080635. 24278298 PMC3836772

[ece373845-bib-0098] Tufail, M. , and M. Takeda . 2008. “Molecular Characteristics of Insect Vitellogenins.” Journal of Insect Physiology 54, no. 12: 1447–1458. 10.1016/j.jinsphys.2008.08.007. 18789336

[ece373845-bib-0099] Visick, O. D. , and F. L. W. Ratnieks . 2023. “Density of Wild Honey Bee, *Apis mellifera* , Colonies Worldwide.” Ecology and Evolution 13, no. 10: e10609. 10.1002/ece3.10609. 37841222 PMC10568204

[ece373845-bib-0100] Wallberg, A. , F. Han , G. Wellhagen , et al. 2014. “A Worldwide Survey of Genome Sequence Variation Provides Insight Into the Evolutionary History of the Honeybee *Apis mellifera* .” Nature Genetics 46, no. 10: 1081–1088. 10.1038/ng.3077. 25151355

[ece373845-bib-0101] Waterhouse, A. M. , J. B. Procter , D. M. A. Martin , M. Clamp , and G. J. Barton . 2009. “Jalview Version 2—A Multiple Sequence Alignment Editor and Analysis Workbench.” Bioinformatics 25, no. 9: 1189–1191. 10.1093/bioinformatics/btp033. 19151095 PMC2672624

[ece373845-bib-0102] Whitfield, C. W. , S. K. Behura , S. H. Berlocher , et al. 2006. “Thrice Out of Africa: Ancient and Recent Expansions of the Honey Bee, *Apis mellifera* .” Science 314, no. 5799: 642–645. 10.1126/science.1132772. 17068261

[ece373845-bib-0103] Wragg, D. , S. E. Eynard , B. Basso , et al. 2022. “Complex Population Structure and Haplotype Patterns in the Western European Honey Bee From Sequencing a Large Panel of Haploid Drones.” Molecular Ecology Resources 22, no. 8: 3068–3086. 10.1111/1755-0998.13665. 35689802 PMC9796960

[ece373845-bib-0104] Wu, B. , Z. Liu , L. Zhou , G. Ji , and A. Yang . 2015. “Molecular Cloning, Expression, Purification and Characterization of Vitellogenin in Scallop Patinopecten Yessoensis With Special Emphasis on Its Antibacterial Activity.” Developmental and Comparative Immunology 49, no. 2: 249–258. 10.1016/j.dci.2014.12.004. 25499034

[ece373845-bib-0105] Zapata‐Hernández, G. , M. Gajardo‐Rojas , M. Calderón‐Seguel , et al. 2024. “Advances and Knowledge Gaps on Climate Change Impacts on Honey Bees and Beekeeping: A Systematic Review.” Global Change Biology 30, no. 3: e17219. 10.1111/gcb.17219. 38450832

[ece373845-bib-0106] Zayed, A. , and C. W. Whitfield . 2008. “A Genome‐Wide Signature of Positive Selection in Ancient and Recent Invasive Expansions of the Honey Bee *Apis mellifera* .” Proceedings of the National Academy of Sciences of the United States of America 105, no. 9: 3421–3426. 10.1073/pnas.0800107105. 18299560 PMC2265178

[ece373845-bib-0107] Zhang, S. , Y. Dong , and P. Cui . 2015. “Vitellogenin Is an Immunocompetent Molecule for Mother and Offspring in Fish.” Fish & Shellfish Immunology 46, no. 2: 710–715. 10.1016/j.fsi.2015.08.011. 26282682

