# Supplementary material for: Population Genetic Study of vitellogenin in Honey Bees ( Apis mellifera ) With European Ancestry Identifies Two Ancestral Genetic Backgrounds
Source: Ecol Evol. 2026 Jun 15;16(6):e73845. doi: 10.1002/ece3.73845 (PMC13269844; doi:10.1002/ece3.73845)
Supplement: Supplementary file 1 — Figure S1: ece373845‐sup‐0001‐supinfo.pdf. Vg polymorphisms, haplotypes, PCoA and explained variance. Figure S2: PCoA of the haplotypes based on common polymorphisms. Figure S3: PCoA of all the haplotypes with putative recombinant sequences labeled. Figure S4: Global F ST calculations. Figure S5: Frequencies of samples with common haplotypes and haplogroups. Figure S6: Frequency of haplogroups per sampling category. Figure S7: Pairwise F ST matrices. Figure S8: Soni et al. (2022) test, private and shared nsSNPs. Figure S9: Pairwise F ST distribution along vg. Figure S10: MKT methods. Table S1: Summary of 65 common polymorphisms. Table S2: Unique number of haplotypes per sampling category and apiary. Table S3: MKT table. Table S4: Location unique nsSNPs summary. Table S5: Location unique deletion summary. [file ECE3-16-e73845-s001.pdf]

Supplement material for:

Population genetic study of vitellogenin in honey bees (*Apis mellifera*) with European ancestry identifies two ancestral genetic backgrounds.

Vilde Leipart, Reed A. Cartwright, Adam Eyre-Walker, Simen R Sandve, and Gro V Amdam

Contents

Supplement Figures — Page

- Figure S1: *Vg* polymorphisms, haplotypes, PCoA and explained variance — 2
- Figure S2: PCoA of the haplotypes based on common polymorphisms — 3
- Figure S3: PCoA of all the haplotypes with putative recombinant sequences labeled — 4
- Figure S4: Global F_{ST} calculations — 5
- Figure S5: Frequencies of samples with common haplotypes and haplogroups — 6
- Figure S6: Frequency of haplogroups per sampling category — 7
- Figure S7: Pairwise F_{ST} matrices — 8
- Figure S8: Soni et al. 2022 test, private and shared nsSNPs — 9
- Figure S9: Pairwise F_{ST} distribution along *vg* — 10
- Figure S10: MKT methods — 11

Supplement Tables

- Table S1: Summary of 65 common polymorphisms — 12
- Table S2: Unique number of haplotypes per sampling category and apiary — 14
- Table S3: MKT table — 14
- Table S4: Location unique nsSNPs summary — 15
- Table S5: Location unique deletion summary — 15

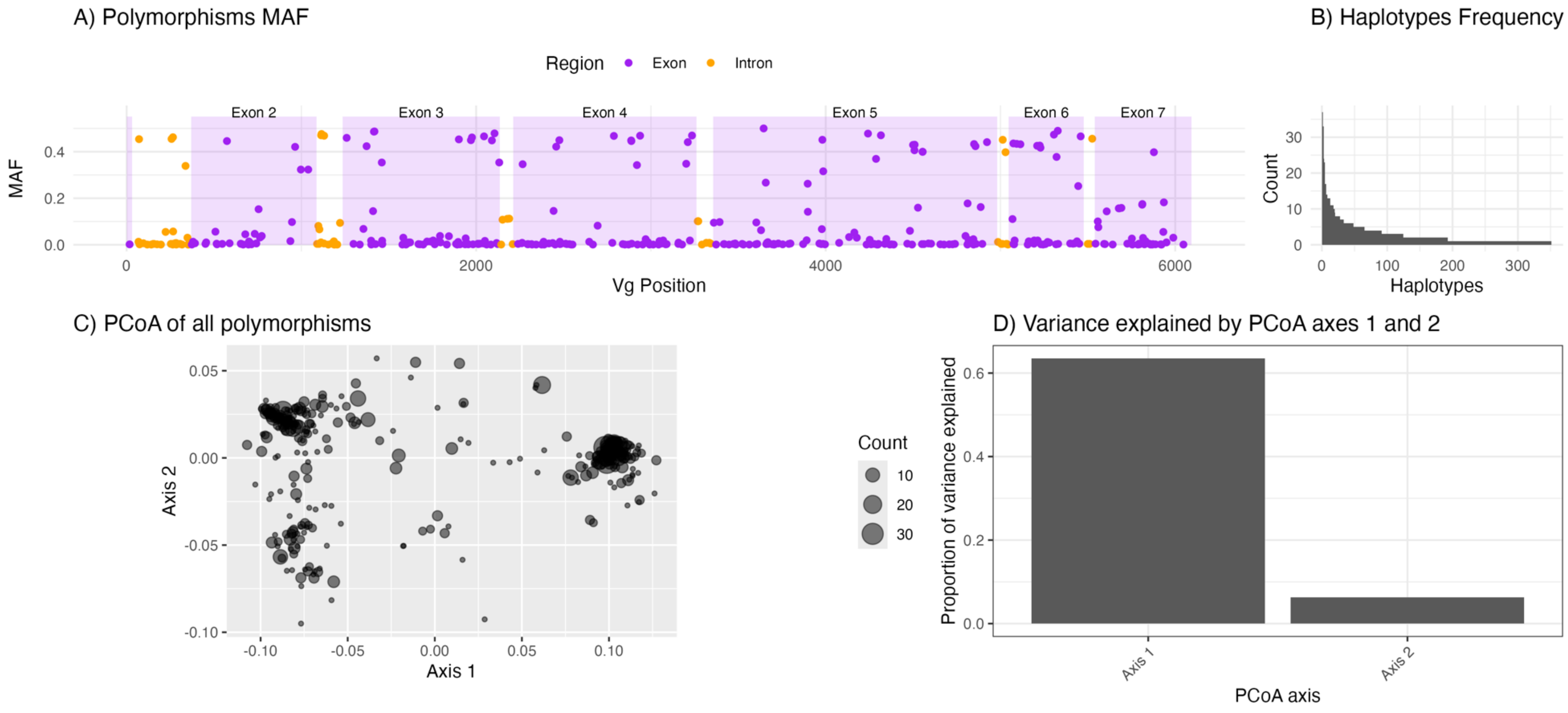

Figure S1. A *Distribution of identified polymorphisms across the vg gene (x-axis) by allele frequency (MAF), colored by intron (yellow) or exon (purple) region.* ***B*** *The identified haplotypes (x-axis) sorted by highest to lowest frequency (y-axis).* ***C*** *Two-dimensional PCoA of the haplotypes identified in panel B, and the abundance of the haplotypes are shown with different-sized spheres. A reference (red), ancestor (blue) and outgroup (green) of the vg gene are included.* ***D*** *Explained variance (y-axis) in the original dataset on the first two axis (x-axis).*

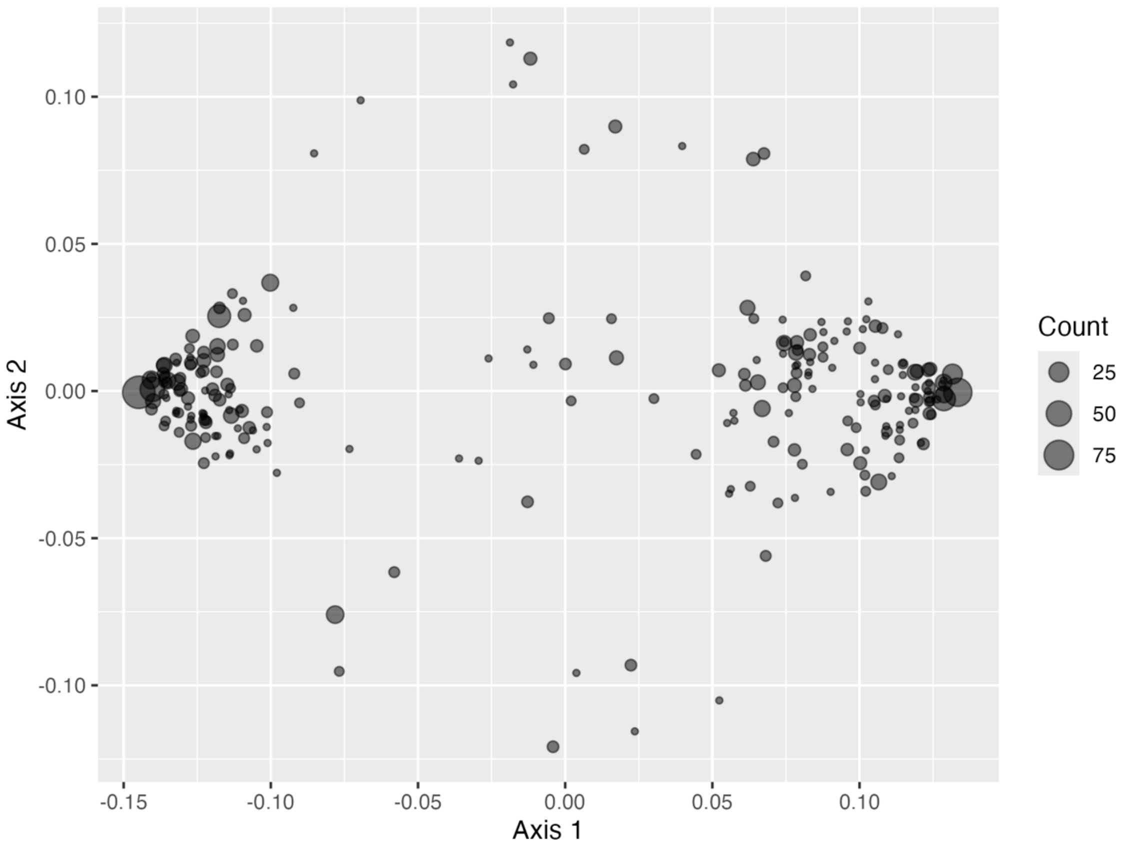

Figure S2: *Two-dimensional PCoA of the haplotypes based on common polymorphisms. The abundance of the unique haplotypes is shown with differently sized circles.*

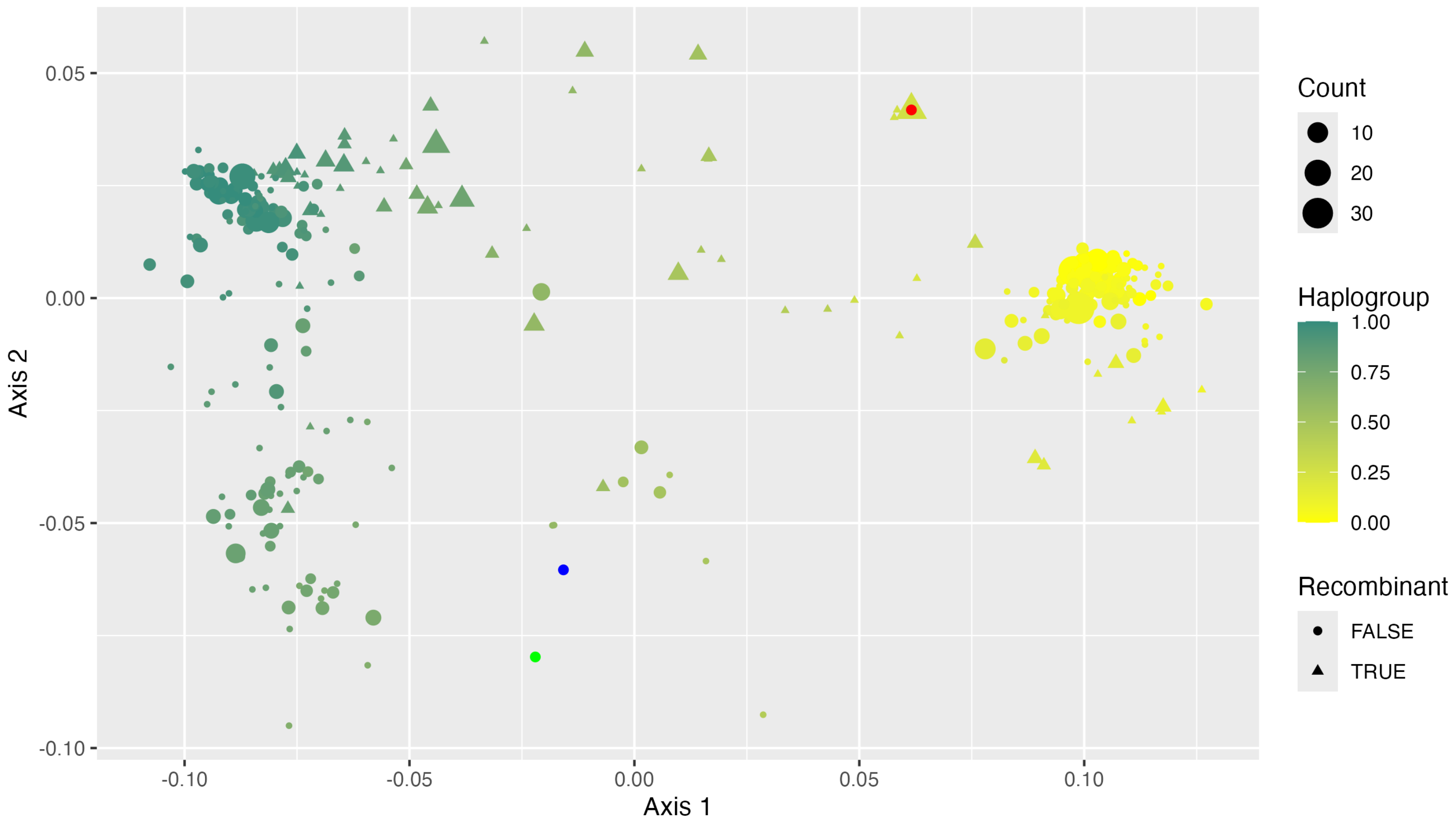

Figure S3*: Two-dimensional PCoA of all the haplotypes identified in Fig. 1, and the abundance of the unique haplotypes is shown with differently sized circles and triangles. The color of the circles and triangles represents the distance to the two most common haplotypes of >0.25 MAF polymorphisms. Green (1) represents the most frequent haplotype (n=95), while brown (-1) represents the second. Recombinant sequences are labeled with triangles and non-recombinant sequences are labeled with circles.*

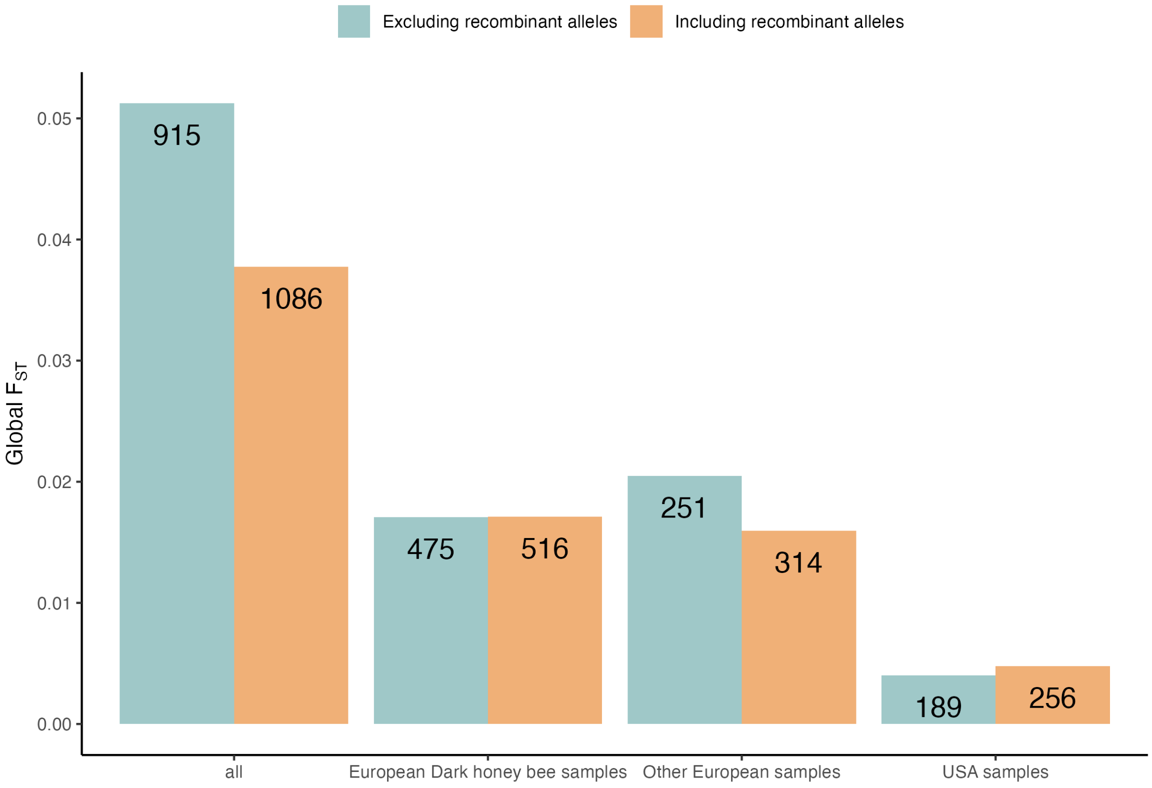

Figure S4. *Global F_{ST} calculated for all sequences and the three geographical regions (x-axis). We calculated the Global F_{ST} excluding recombinant haplotypes (teal) and using all haplotypes (including recombinant, orange). The sample size per group is labeled in each bar.*

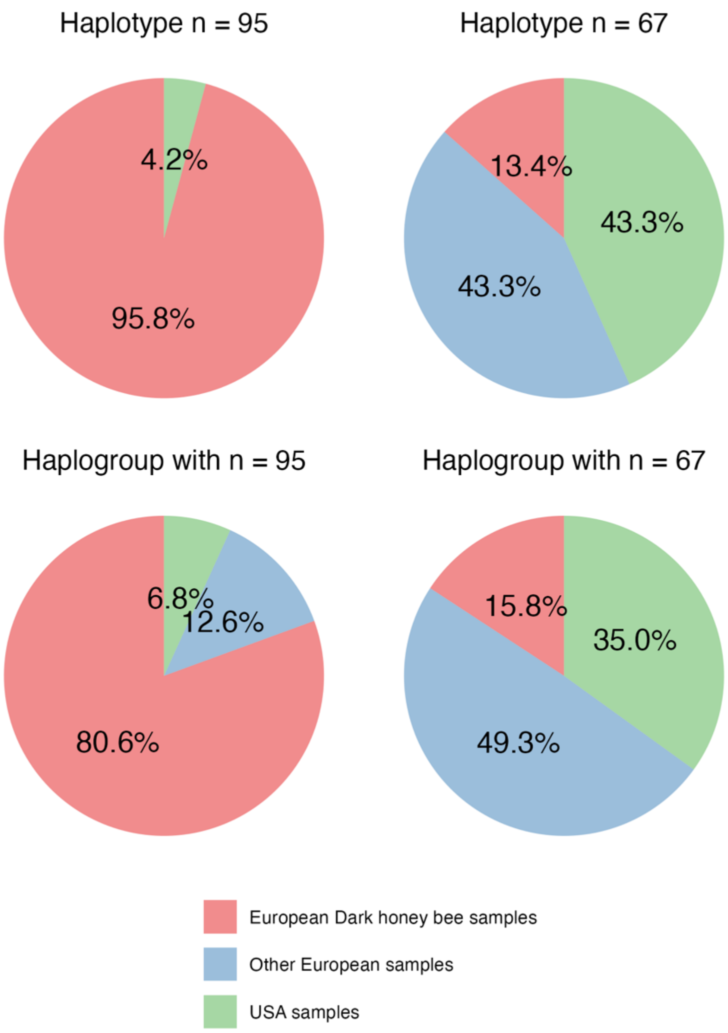

Figure S5: *Pie charts showing the calculations of the percentage of samples from each sampling category with the two most common haplotypes (n=95, n=67, top row) and within haplogroups 1 and 2 (closely related to n = 95 and n = 67, lower row). The top row only uses samples with haplotype n = 95 or n = 67. The lower row includes samples with a distance < 0.2 to the reference haplotype n = 95 or n = 67 (See methods for details on distance calculations using reference haplotypes).*

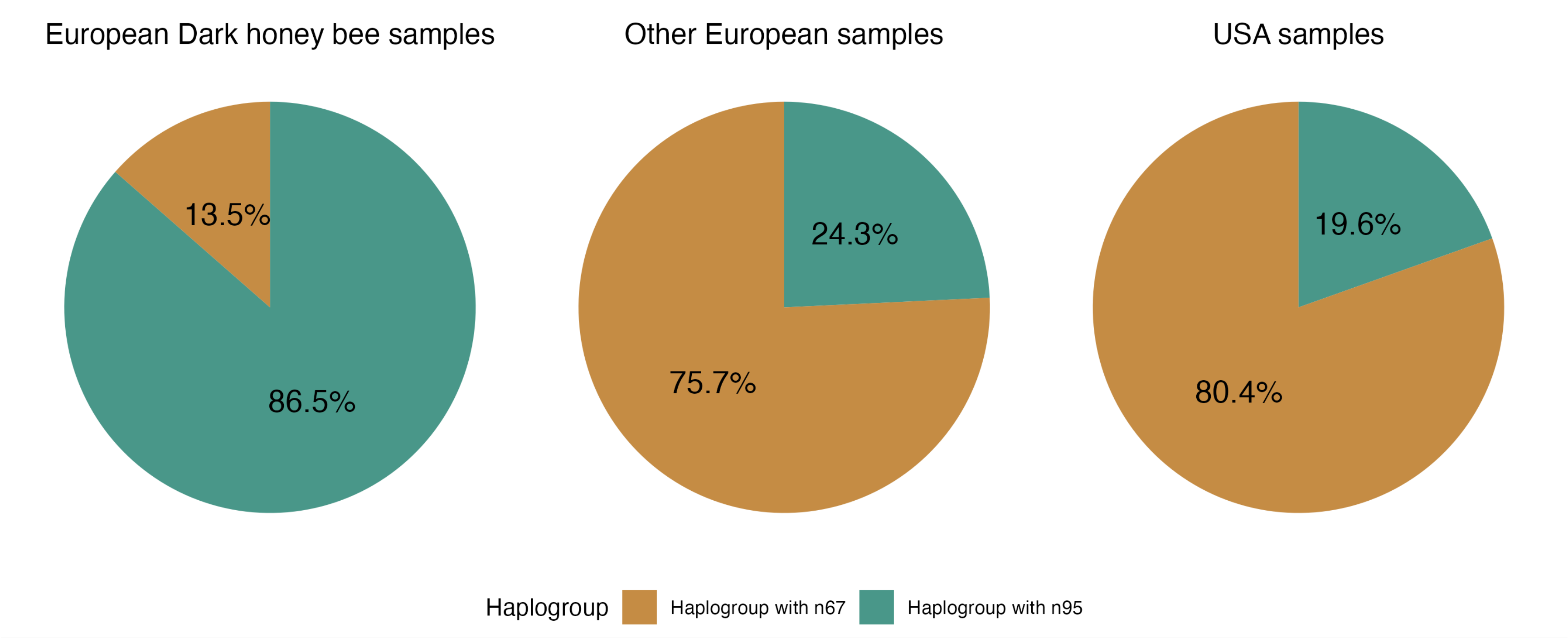

Figure S6: *Pie charts showing the calculations of the percentage of haplogroup 1 (n = 95) and 2 (n = 67) per sampling category.*

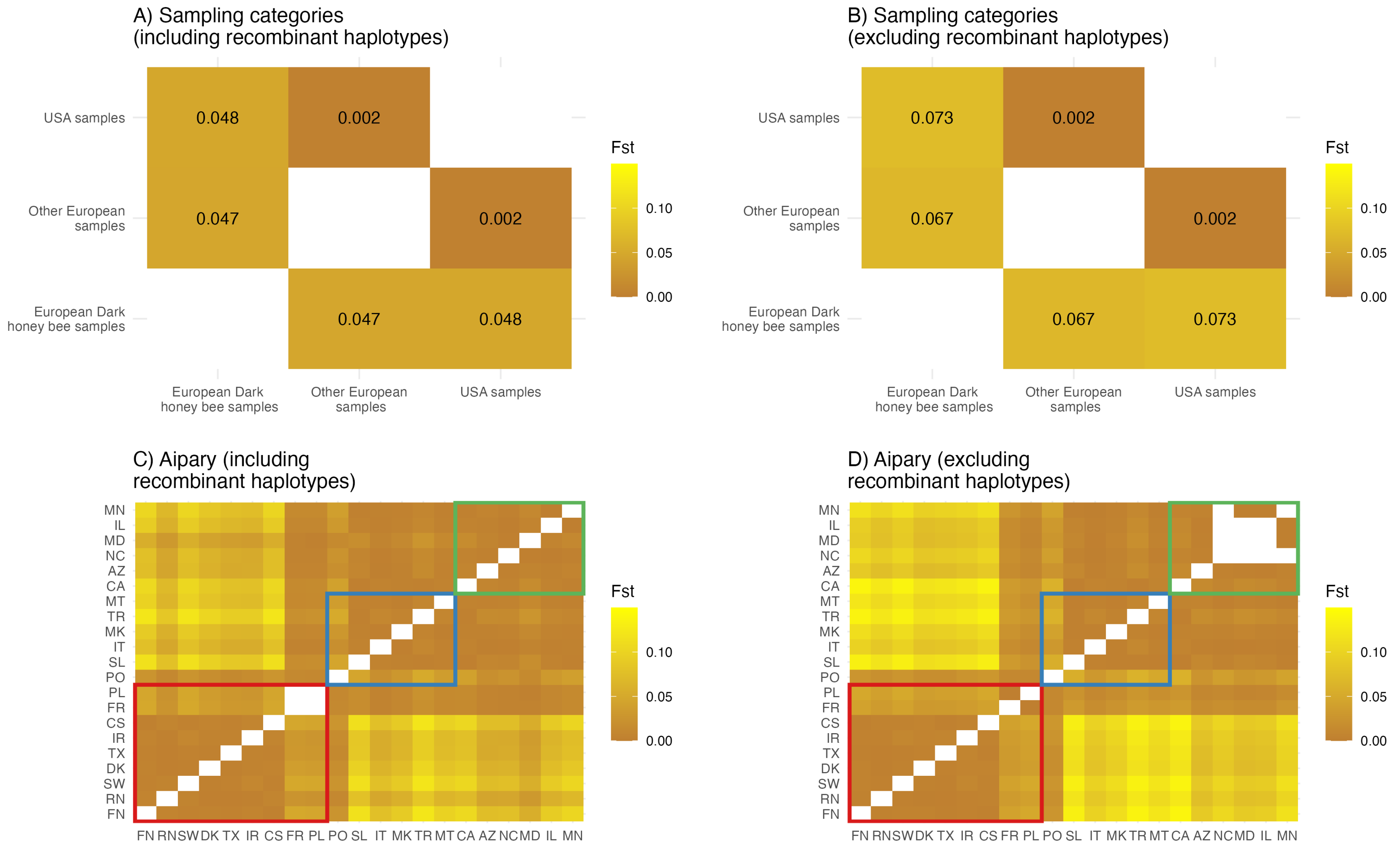

Figure S7: *Heatmaps of pairwise F_{ST}.* ***A*** *The three sampling categories including recombinant haplotypes,* ***B*** *same as panel A, but excluding recombinant haplotypes.* ***C*** *All apiaries including recombinant haplotypes, with a red box labeling the European Dark honey bee samples, blue box for the other European samples and green for the USA samples,* ***D*** *same as panel C, but excluding recombinant haplotypes.*

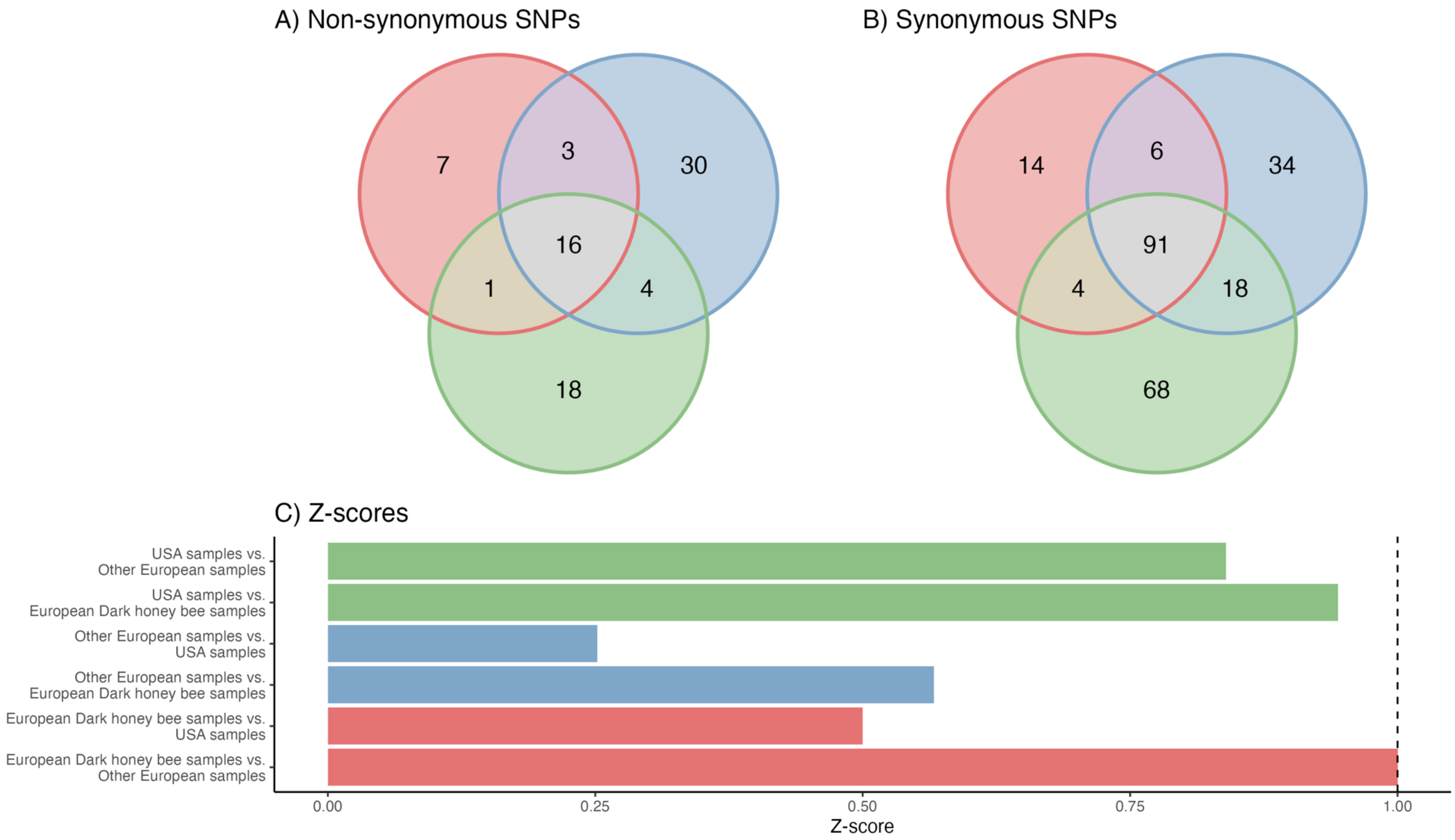

Figure S8: ***A*** *Venn diagram of private and shared nsSNPs. The circles are colored by sampling categories: European Dark honey bee samples (red), other European samples (blue) and USA samples (green).* ***B*** *Same as in panel A, but counting the synonymous SNPs.* ***C*** *Histogram of Z values (x-axis). On the y-axis are the comparisons. A dotted line on Z = 1, which is considered the threshold for sign of balancing selection.*

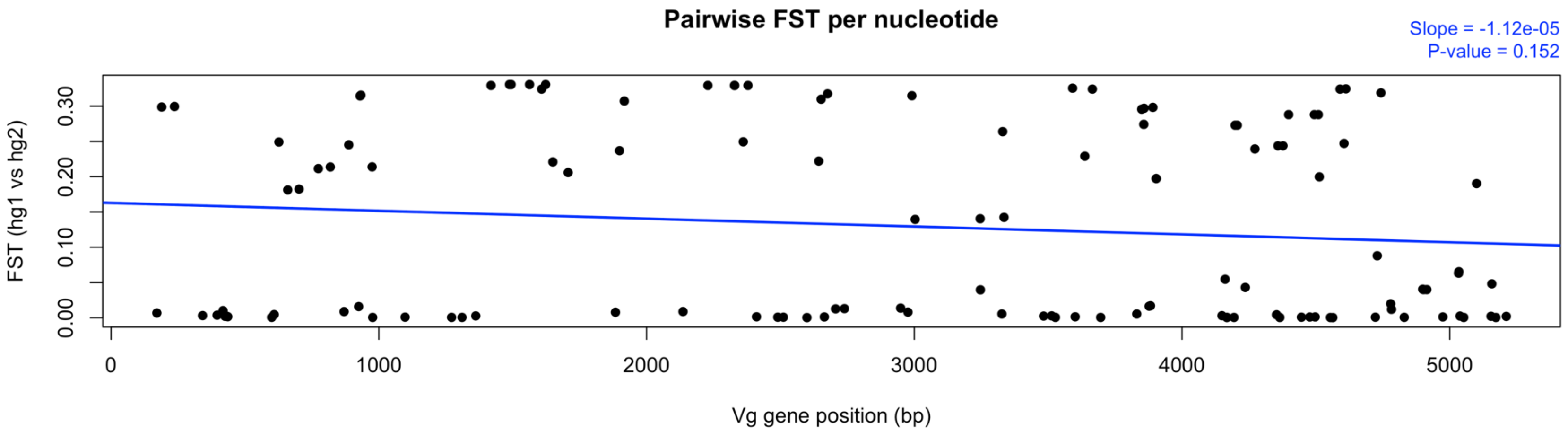

***Figure S9**: Plotting the pairwise F_{ST} for all positions along the vg gene between haplogroups 1 and 2 (hg1 and hg2, the haplogroups are defined as described in methods and shown in Fig 1, sequences with the two most common haplotypes, plus any haplotype with a pairwise distance < 0.2, y-axis) along the gene (x-axis). The blue line represents the linear regression, which has a slight negative slope (see blue text on top of the plot), but this is not significant.*

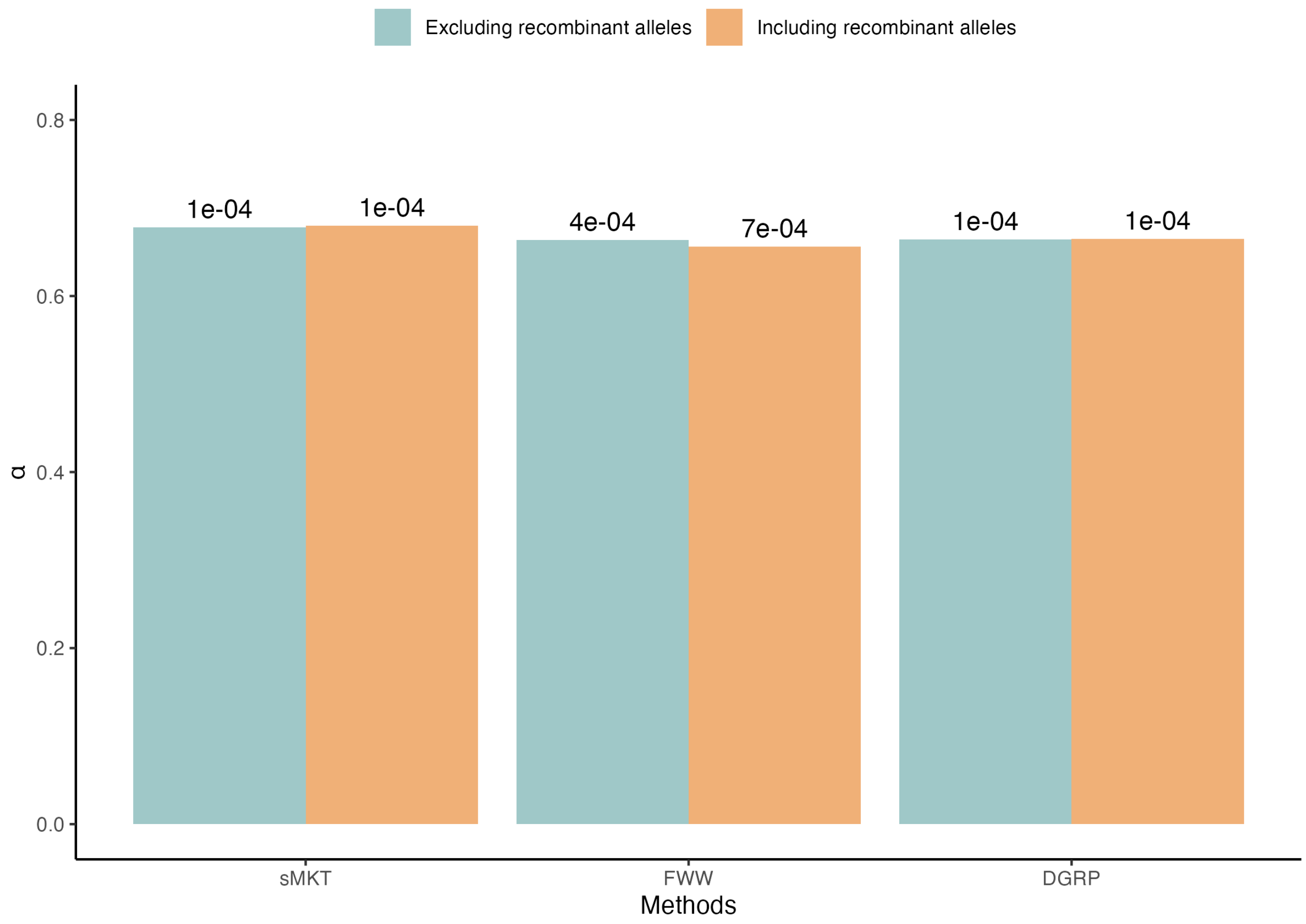

Figure S10: *Estimated fraction of nsSNPs driven by positive selection (y-axis) using three different MK tests(x-axis): Standard MKT (sMKT), corrected MKT (FWW) and extended MKT (DGRP). The p-value is labeled on top of each bar.*

Table S1: *The 65 polymorphisms separating the haplotypes. For each polymorphism, we report cDNA position, coding sequence (CDS) position, minor allele frequency (MAF), gene region (intron or exon), amino acid position (Pos aa), and Vg domain.*

Pos DNA	Pos CDS	MAF	Region	Ref nt	Alt nt	Pos aa	Ref aa	Alt aa	Vg domain
71	*non-coding*	0.454	Intron 1	A	G	*non-coding*			
257	*non-coding*	0.4549	Intron 1	C	T	*non-coding*			
265	*non-coding*	0.4622	Intron 1	C	T	*non-coding*			
337	*non-coding*	0.3389	Intron 1	A	-	*non-coding*			
574	237	0.4457	Exon 2	A	G	79	Q	Q	β-barrel
964	627	0.4208	Exon 2	C	T	209	R	R	β-barrel
997	660	0.3232	Exon 2	T	C	220	D	D	β-barrel
1039	702	0.3232	Exon 2	T	C	234	D	D	β-barrel
1113	*non-coding*	0.4705	Intron 2	A	G	*non-coding*			
1115	*non-coding*	0.4761	Intron 2	T	C	*non-coding*			
1130	*non-coding*	0.4687	Intron 2	A	G	*non-coding*			
1259	774	0.4595	Exon 3	C	T	258	V	V	β-barrel
1373	888	0.4236	Exon 3	C	T	296	N	N	β-barrel
1415	930	0.4862	Exon 3	C	T	310	P	P	β-barrel
1418	933	0.4871	Exon 3	G	A	311	T	T	β-barrel
1460	975	0.3536	Exon 3	T	C	325	Y	Y	β-barrel
1901	1416	0.453	Exon 3	C	T	472	N	N	α-helical
1970	1485	0.4494	Exon 3	G	A	495	T	T	α-helical
1976	1491	0.4613	Exon 3	C	T	497	F	F	α-helical
2045	1560	0.4659	Exon 3	G	A	520	G	G	α-helical
2090	1605	0.4484	Exon 3	G	A	535	P	P	α-helical
2105	1620	0.4788	Exon 3	A	G	540	T	T	α-helical
2132	1647	0.3536	Exon 3	C	T	549	F	F	α-helical
2266	1704	0.3462	Exon 4	A	C	568	A	A	α-helical
2458	1896	0.4217	Exon 4	T	C	632	T	T	α-helical
2476	1914	0.4494	Exon 4	C	A	638	G	G	α-helical
2788	2226	0.4678	Exon 4	T	C	742	S	S	α-helical
2887	2325	0.4484	Exon 4	A	T	775	S	S	lipid-binding cavity
2888	2326	0.4457	Exon 4	A	C	776	R	R	lipid-binding cavity
2920	2358	0.3425	Exon 4	C	T	786	N	N	lipid-binding cavity
2938	2376	0.4687	Exon 4	T	C	792	H	H	lipid-binding cavity

3202	2640	0.3481	Exon 4	C	T	880	N	N	lipid-binding cavity
3211	2649	0.4411	Exon 4	G	C	883	A	A	lipid-binding cavity
3235	2673	0.4696	Exon 4	A	G	891	K	K	lipid-binding cavity
3645	2988	0.5	Exon 5	T	C	996	D	D	lipid-binding cavity
3657	3000	0.267	Exon 5	C	T	1000	H	H	lipid-binding cavity
3897	3240	0.2624	Exon 5	G	A	1080	R	R	lipid-binding cavity
3981	3324	0.4512	Exon 5	T	C	1108	P	P	lipid-binding cavity
3986	3329	0.3158	Exon 5	A	G	**1110**	**S**	**T**	lipid-binding cavity
4242	3585	0.4779	Exon 5	T	A	1195	V	V	lipid-binding cavity
4288	3631	0.3692	Exon 5	C	T	1211	L	L	lipid-binding cavity
4316	3659	0.4705	Exon 5	A	G	**1220**	**N**	**S**	lipid-binding cavity
4500	3843	0.4291	Exon 5	G	A	1281	K	K	lipid-binding cavity
4508	3851	0.4061	Exon 5	G	A	**1284**	**R**	**K**	lipid-binding cavity
4509	3852	0.43	Exon 5	G	A	1284	R	R	lipid-binding cavity
4554	3897	0.3996	Exon 5	A	G	1299	E	E	lipid-binding cavity
4555	3898	0.3996	Exon 5	T	C	1300	L	L	lipid-binding cavity
4849	4192	0.4328	Exon 5	A	G	**1398**	**I**	**V**	lipid-binding cavity
4857	4200	0.4236	Exon 5	C	T	1400	N	N	lipid-binding cavity
4923	4266	0.4411	Exon 5	T	C	1422	D	D	lipid-binding cavity
5013	*non-coding*	0.4512	Intron 6	G	A	*non-coding*			
5029	*non-coding*	0.3978	Intron 6	C	T	*non-coding*			
5074	4352	0.4355	Exon 6	T	C	**1451**	**V**	**A**	vWF domain
5093	4371	0.4337	Exon 6	A	G	1457	K	K	vWF domain
5114	4392	0.4319	Exon 6	C	T	1464	G	G	vWF domain
5210	4488	0.4254	Exon 6	C	T	1496	V	V	vWF domain
5225	4503	0.4263	Exon 6	C	T	1501	N	N	vWF domain
5229	4507	0.4171	Exon 6	A	G	**1503**	**T**	**A**	vWF domain
5306	4584	0.4733	Exon 6	G	A	1528	E	E	vWF domain
5321	4599	0.3775	Exon 6	T	A	1533	G	G	vWF domain
5328	4606	0.4899	Exon 6	A	G	**1536**	**I**	**V**	vWF domain
5445	4723	0.2523	Exon 6	C	T	1575	L	L	vWF domain
5459	4737	0.4659	Exon 6	T	G	1579	G	G	vWF domain
5525	*non-coding*	0.4558	Intron 6	A	G	*non-coding*			
5878	5094	0.3978	Exon 7	T	C	1698	D	D	C-terminal

Table S2: *Unique haplotypes observed per sampling category and apiary. We also report how many of these unique haplotypes that are considered haplogroup 1 or haplogroup 2 (column 4 and 5).*

Sampling category	Apiaries	Unique haplotypes	Haplogroup 1	Haplogroup 2
**European Dark honey bee samples**	FN	15	15	0
	RN	22	16	4
	SW	9	9	0
	DK	15	13	1
	TX	15	10	4
	CS	10	10	0
	IR	13	10	1
	FR	18	7	7
	PL	18	4	10
**Other European samples**	PO	37	24	11
	IT	27	12	14
	MT	26	2	17
	MK	38	6	24
	SL	25	3	18
	TR	27	1	22
**USA samples**	AZ	20	3	9
	CA	17	1	10
	IL	16	3	9
	MD	16	4	7
	MN	19	4	12
	NC	20	3	10

Table S3*: MKT table of the neutral and selected polymorphisms, and whether they are divergent.*

All haplotypes (excluding recombinant haplotypes)	Polymorphism, P	Divergence, D
Neutral class, S	41 (43)	117 (118)
Selected class, N	22 (23)	196 (196)

Table S4: *nsSNPs and their allele frequency identified in apiaries and/or subspecies. Bold if the MAF is >0.1.*

Category	Apiaries	Subspecies	nsSNPs	MAF
European Dark honey bee samples	FN and RN	*A. m. mellifera*	p.P866S, p.D1103Y	0.085, 0.051
	FN and DK		p.V942A	0.052
	RN		**p.T1567M**	**0.125**
	IR		p.A1237V	0.019
	FR		p.E906K, **p.M1559I**	0.018, **0.107**
	PL		p.G1016S	0.071
Other European samples	MT	*A. m. ruttneri*	p.A60T, p.T984M, p.G1565S, p.S1713F	0.089, 0.071, 0.089, 0.018
	PO	*A. m. iberiensis*	p.T594M, p.V661M, p.L1072F	0.017, 0.017, 0.052
	IT	*A. m. ligustica*	p.L606F, p.T1207I	0.068, 0.091
	SL	*A. m. carnica*	p.L828I	0.020
	TR	*A. m. anatolica*	p.P958S, p.G1016D, p.V1508M, p.P1755S	0.024, 0.024, 0.024, 0.024
	MK	*A. m. macedonica*	p.V1397M	0.016
USA samples	MD and NC		p.P126L	0.023
	AZ and IL		p.I640V, p.T1567K, p.L1670S	0.036, 0.071, 0.060
	AZ		p.S467N, **p.I489V**, p.M525I, p.N560H, p.T1110A, p.V1193L, p.R1385K, p.P1620S	0.100, **0.125**, 0.025, 0.100, 0.100, 0.025, 0.025, 0.025
	CA		p.R969H	0.025
	IL		p.G1504R	0.045
	NC		p.I943M, p.T1692I	0.023, 0.023
	MD		p.T1013M	0.045

Table S5: *Deletions and their allele frequency identified in apiaries and/or subspecies. Bold if the MAF is >0.1.*

Category	Apiaries	Subspecies	Deletion	MAF
European Dark honey bee samples	RN, FN, SW, DK, TX, SC, IR, PL	*A. m. mellifera*	**p.N153_V155del**	**0.202**
Other European samples	SL	*A. m. carnica*	p.N153_V155del	0.020
	MK	*A. m. macedonica*	p.N153_V155del	0.016
USA samples	CA, MN, NC and MD		p.N153_V155del	0.068
	IL		p.S844_V845del	0.068
	MD		p.R1669del	0.023
